# Investigation of Antimicrobial Activity of Amino Acid- and Triphenylphosphonium-Based Derivatives of Chrysin and Pinocembrin

**DOI:** 10.3390/antibiotics15101025

**Published:** 2026-10-15

**Authors:** Jeslin Cheruvathoor Jenny, Andrzej S. Skwarecki, Michał K. Pierański, Katarzyna Kozłowska-Tylingo, Piotr Szweda

**Affiliations:** Department of Pharmaceutical Technology and Biochemistry, Gdańsk University of Technology, 11/12 Gabriela Narutowicza Street, 80-233 Gdańsk, Poland; michal.pieranski@pg.edu.pl (M.K.P.); katarzyna.kozlowska-tylingo@pg.edu.pl (K.K.-T.); piotr.szweda@pg.edu.pl (P.S.)

**Keywords:** propolis, flavonoids, pinocembrin, chrysin, *Candida*

## Abstract

Objectives: Chrysin and pinocembrin are two key flavonoids contributing to the antimicrobial activity of poplar propolis. However, their antimicrobial potency when used independently is limited. In this study, amino acid (AA)- and triphenylphosphonium (TPP)-based derivatives of chrysin and pinocembrin were designed, synthesised, and evaluated for their antimicrobial activity, with particular focus on their antifungal potential and mechanism of action. Methods: AA- and TPP-based derivatives of chrysin and pinocembrin were synthesised and characterised. Their antimicrobial activity was evaluated against selected bacterial and fungal strains. The mechanism of antifungal activity of the most active compounds was further investigated. Additionally, their haemolytic activity was also evaluated. Results and Conclusions: TPP derivatives, compounds **11** and **12**, showed substantially enhanced antimicrobial activity against *Candida albicans*, *Candida glabrata*, *Candida krusei*, *Staphylococcus aureus*, and *Escherichia coli*. Further investigation indicated that compounds **11** and **12** are capable of inducing membrane depolarisation while exhibiting negligible haemolytic activity. These findings support TPP-based derivatisation as a promising approach for improving the antimicrobial potential of these propolis-derived flavonoids.

## 1. Introduction

**Figure 1:**
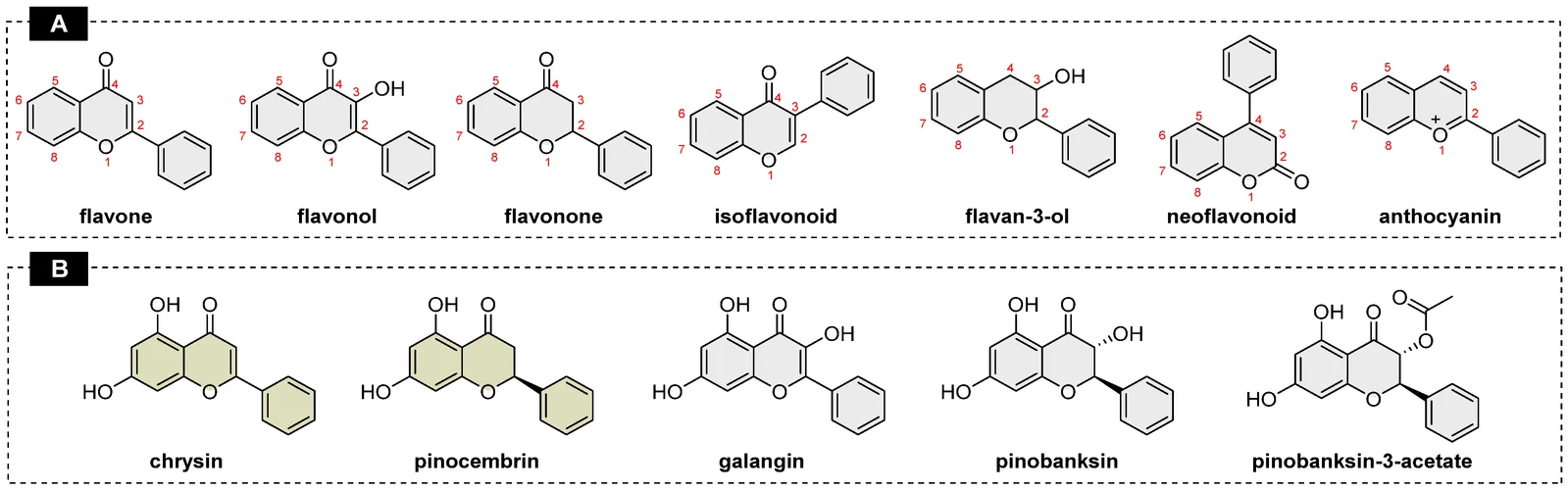
(**A**) General scaffolds of naturally occurring flavonoids. (**B**) Chemical structures of flavonoids with proved antifungal activity.

Flavonoids are plant-derived secondary metabolites produced in response to environmental stress and possess diverse therapeutic and pharmacological activities [1]. More than 9000 compounds are identified as flavonoids with a characteristic three-ring skeleton (C6-C3-C6) and are classified into subclasses—flavones, flavonols, flavanones, isoflavonoids, neoflavonoids, flavanols and anthocyanins (Figure 1), based on their molecular structure [2,3]. Various studies have confirmed the antioxidant, antiviral, antimicrobial, antidiabetic, anticancer, anti-inflammatory, neuroprotective and cardioprotective potency of these flavonoids [1,4]. This family of hydroxylated phenolic compounds contributes to the bioactive fraction of various natural products [5]. Besides plants, a wide variety of flavonoids are also present in propolis along with other types of plant-based organic compounds.

Propolis is a plant-sourced resinous matrix produced by honeybees to protect, thermally insulate and mechanically strengthen their hive [6]. It was popular in traditional medicine. Various studies have confirmed its antimicrobial, anti-inflammatory, anti-ulcer, antitumor, antioxidant and wound-healing properties [7]. In a recent study, flavonoids like pinocembrin, pinobanksin-3-acetate, chrysin, galangin, pinobanksin, techtochrysin, genkwanin, pinostrobin and sakuranetin isomer were statistically identified as key contributors to the antifungal activity in hydroethanolic extracts of propolis [8]. Independent use of these compounds failed to inhibit the targeted microbial growth, suggesting the possibility of a synergistic effect of multiple components to yield the desired activity. Further, a combination of five compounds: chrysin, galangin, pinocembrin, pinobanksin, and pinobanksin-3-acetate was formulated with high antifungal potency [8]. However, the low concentration of flavonoids in its natural source and complex extraction and isolation processes make the availability of these compounds scarce [9]. Herein, we focus on two flavonoids; chrysin and pinocembrin, identified as crucial for the antifungal potency of propolis. These flavonoids are characteristic of poplar propolis [6].

Chrysin (5,7-dihydroxyflavone) and pinocembrin (5,7-dihydroxyflavanone) are structurally related flavonoids with a difference in the degree of C2-C3 saturation. The presence of a C2=C3 double bond in chrysin results in a conjugated and planar molecular structure, which enhances its lipophilic character relative to pinocembrin, whose C2–C3 bond is saturated. Generally, flavonoids show low therapeutic efficacy and bioavailability due to poor absorption and rapid metabolism leading to rapid elimination [10]. The lipophilic nature of these molecules makes them poorly soluble in water, thereby reducing their bioavailability [11]. Chrysin and pinocembrin, like many other flavonoids, hence demand structural modification which can activate or improve their antimicrobial potency. Numerous derivatisation strategies including etherification, esterification, transition-metal-mediated couplings, sigmatropic rearrangements, and electrophilic substitutions, have been reported to improve the bioactivity of chrysin [12]. Herein, two approaches—amino acid (AA)-based derivatisation and triphenylphosphonium-based derivatisation of chrysin and pinocembrin—are adopted to study the change in antimicrobial activity resulting from these modifications.

AA conjugation represents a well-established strategy for improving the physicochemical and pharmacokinetic properties of bioactive compounds. AA-based prodrugs can improve the water solubility and permeability of flavonoids. Prodrugs are derivatives of active ingredients which undergo chemical or enzymatic transformation to release the active compound [13]. The nature of the AA largely depends on the structure of its side chain, which can be nonpolar, polar-uncharged, or charged. Most AA prodrugs are prepared by forming an ester or amide linkage between the α-amino or α-carboxyl group of the amino acid and the complementary functional group on the parent drug molecule. Amide bonds are more stable than ester bonds, while carbonate and carbamate links are also possible between the molecules. The increase in water solubility of AA prodrugs is primarily a result of the addition of an ammonium cation or carboxylate anion to the molecule. Formation of a salt of the prodrug can further improve the solubility [14]. AAs are zwitterionic molecules that cease to exist in their zwitterionic form upon prodrug formation, unless they possess additional functional groups in their R group. The aqueous solubility of AAs depends largely on the polarity and ionisation of their R groups. AAs bearing nonpolar R groups generally exhibit lower water solubility than those containing polar or charged side chains. Consequently, the nature of the conjugated amino acid can significantly influence the aqueous solubility of the resulting conjugates [13]. Development of AA conjugates accounts for their stability, with high stability for better shelf life while not overlooking the ease of release of the drug molecule under ambient conditions [15]. Ester bonds are more labile to chemical and enzymatic hydrolysis than other types of bonds [14]. Formation of an ester bond with AA on the 7-OH can improve the solubility while keeping the rest of the molecule unaltered, protecting the pharmacophore. In this study, we aim to synthesise AA ester conjugates of chrysin and pinocembrin, as these can be easily hydrolysed under ambient conditions. The amino acids selected are L-lysine, bearing a positively charged and hydrophilic side chain; L-serine, bearing a polar uncharged side chain; and glycine, L-leucine, and L-proline, representing amino acids with neutral side chains of varying hydrophobicity.

In parallel, triphenylphosphonium (TPP)-based conjugates of flavonoids were investigated in this work as another approach to improve the bioavailability of flavonoids. TPP derivatives have gained traction in drug design and delivery as a mitochondrial-targeted cationic conjugate. Structurally, it contains cationic phosphorus bonded to three phenyl groups and a variable R group. The cationic phosphorous, along with hydrophobic phenyl rings, can facilitate cell membrane and mitochondrial membrane permeation [16]. The membrane potential of bacterial cells ranges from −100 to −200 mV, while that of eukaryotic cells ranges from −60 to −80 mV. This potential gradient drives the cationic TPP to target the higher negative potential. The same principle is true for fungi, protozoa and parasites [17]. The TPP cation is bonded to the drug molecule through a linker molecule. A longer alkyl chain is generally preferred as the TPP-alkyl moiety, with more than 10 carbons possessing antimicrobial activity by itself. On the contrary, a short alkyl linker uses the TPP moiety to target the parent molecule to the targeted cells without exerting any activity by itself [17]. Ether bonds are more stable than ester bonds and can improve the lipophilicity of the drug molecule. Based on this rationale, novel TPP-conjugated chrysin and pinocembrin derivatives were designed and synthesised by linking the flavonoid core to the TPP moiety via a three-carbon alkyl spacer through an ether bond, with the aim of enhancing cellular uptake and antimicrobial activity.

In this study AA-flavonoids and TPP-flavonoids are synthesised, and their antimicrobial activity is evaluated. Further insights into the mechanisms underlying their antifungal activity were also investigated. Successful structural modification of chrysin and pinocembrin, if translated into efficient antimicrobial activity, could provide a platform for its broadening to other flavonoids as well.

## 2. Results and Discussion

### 2.1. Chemistry

**Figure 2:**
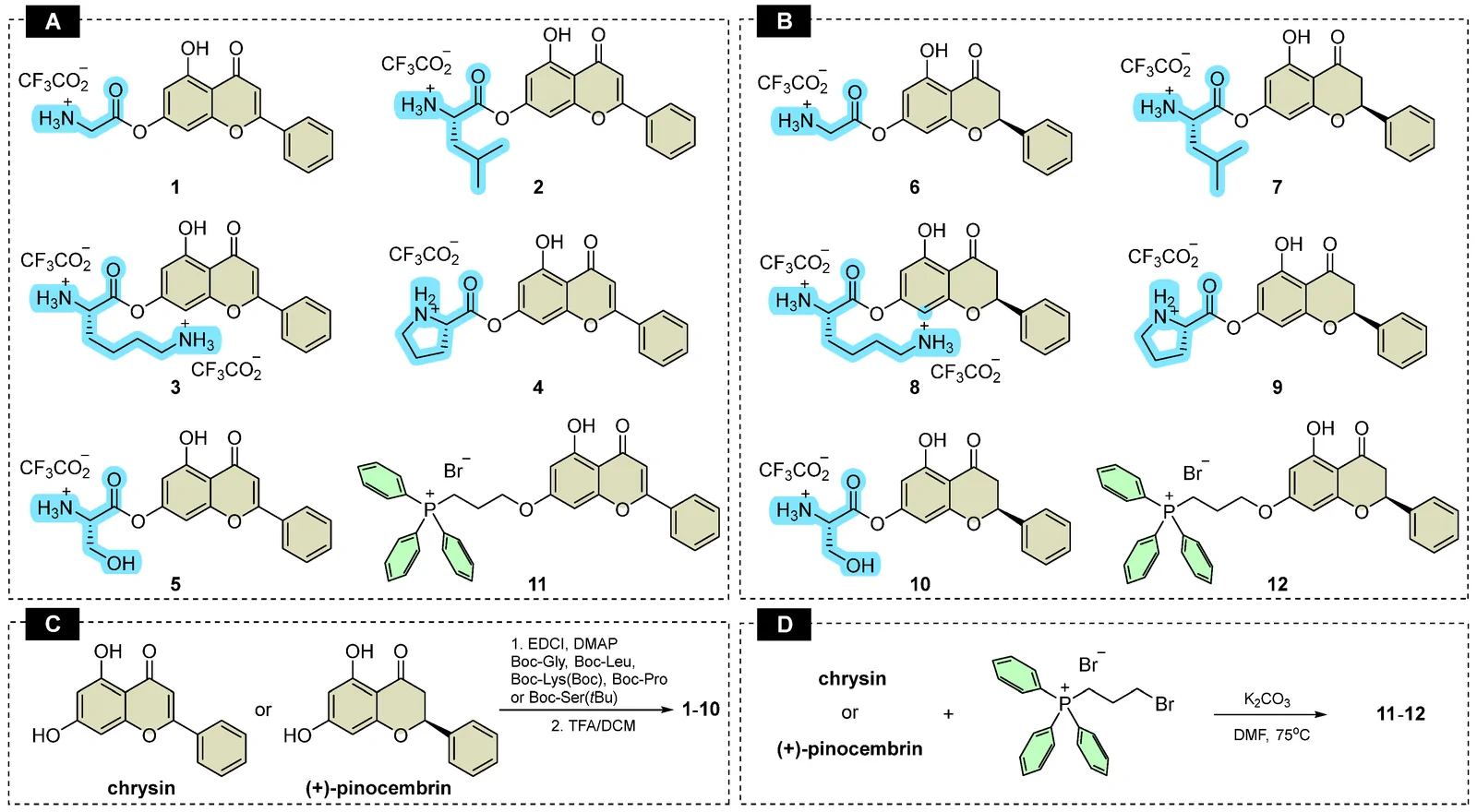
(**A**) Chemical structures of the prepared chrysin-based conjugates. (**B**) Chemical structures of the prepared pinocembrin-based conjugates. (**C**) Synthesis of the chrysin and pinocembrin conjugates with amino acids. (**D**) Synthesis of the chrysin and pinocembrin with triphenylphosphonium (TPP^+^) cation.

Conjugates **1**–**10** (Figure 2A,B) were synthesised by mild Steglich esterification of commercially available chrysin and pinocembrin with Boc-protected amino acids using EDCI and DMAP as coupling reagents (Figure 2C). The esterification step was followed by removal of the Boc protecting group under acidic conditions using trifluoroacetic acid (TFA). In the case of the serine derivatives, the *tert*-butyl ether protecting group was removed simultaneously. The ^1^H NMR spectra of the isolated conjugates were consistent with the formation of the desired products. The regioselectivity of the conjugation was confirmed by ^1^H NMR spectroscopy. The characteristic strongly downfield-shifted signal of the 5-OH proton, arising from intramolecular hydrogen bonding with the C4 carbonyl group, remained present in all conjugates at approximately δ 13 ppm for the chrysin derivatives and δ 12 ppm for the pinocembrin derivatives. This confirms that the 5-OH group remained unsubstituted and that conjugation occurred at the 7-OH position.

Interestingly, the ^13^C NMR spectra of compounds **1**, **3**, **5**, and **8** contained additional signals attributable to the corresponding parent flavonoids and amino acids, although no evidence of these species was observed in the ^1^H NMR spectra recorded shortly after sample preparation. This discrepancy suggests that partial cleavage of the ester linkage may have occurred in situ during the considerably longer acquisition of the ^13^C NMR spectra. Such degradation could result from hydrolysis in the presence of residual water or, potentially, from intramolecular cleavage promoted by nucleophilic functionalities present in the conjugates. Consistent with this interpretation, the additional ^13^C NMR signals included resonances in the carboxyl carbon region (approximately 170 ppm) attributable to the released amino acid components, together with signals corresponding to the respective parent flavonoids. These observations indicate that some of the amino acid–flavonoid ester conjugates are susceptible to gradual cleavage under the conditions used for NMR analysis.

The TPP-based conjugates **11** and **12** (Figure 2A,B) were synthesised in a single step via Williamson ether synthesis using commercially available 3-(bromopropyl)triphenylphosphonium bromide (Figure 2D). The structures of both conjugates were confirmed by ^1^H and ^13^C NMR spectroscopy.

### 2.2. LC-MS Analysis of the Obtained Compounds

LC–MS analysis of the synthesised compounds further confirmed the rapid hydrolysis of amino acid conjugates **1**–**10**. Figures S25 and S26 show the chromatograms of the chrysin- and pinocembrin-based derivatives, respectively, with individual peaks assigned on the basis of ESI-MS data. Under the applied chromatographic conditions, chrysin and pinocembrin exhibited retention times of 17.0 and 17.1 min, respectively, whereas the more hydrophilic amino acid conjugates eluted at shorter retention times. Among the investigated derivatives, the peak area of leucine conjugates of both flavonoids showed the lowest extent of hydrolysis under the applied elution conditions, suggesting their comparatively higher stability in aqueous media. ESI-MS analysis confirmed the presence of the molecular ions corresponding to the desired conjugates in the respective samples. In addition, ions corresponding to the parent flavonoids and the respective amino acids were detected, providing further evidence of hydrolytic cleavage of the amino acid conjugates. As the amino acids used in this study lack suitable chromophores for DAD detection under the applied conditions, they were not observed in the corresponding DAD chromatograms.

The susceptibility of these conjugates to hydrolysis was particularly evident for Gly-Chrysin (**1**). A parallel analysis of compound **1** using an H_2_O/ACN mobile phase resulted in complete conversion of the conjugate, with only a single chromatographic peak corresponding to chrysin (17.0 min). In contrast, when the same compound was subsequently analysed using the initial mobile-phase system, two peaks corresponding to Gly-chrysin (**1**) and free chrysin were observed. These results demonstrate the pronounced susceptibility of compound **1** to hydrolysis in an aqueous environment.

Ester hydrolysis can be strongly influenced by the pH of the medium and the structural features of the amino acid moiety. In α-amino acid esters, the protonated α-amino group exerts an electron-withdrawing effect that may increase the susceptibility of the ester linkage to hydrolysis [18]. Depending on the structure of the substrate and the reaction conditions, hydrolysis of amino acid esters may proceed through several pathways, including intramolecular general-base or nucleophilic catalysis, general acid–base catalysis, and specific base catalysis [18]. In the present series, the chromatographic peak area of the leucine derivatives **2** and **7** indicated comparatively lower levels of hydrolysis, which may be associated with steric shielding provided by the branched aliphatic side chain. In contrast, the glycine derivative **1** and proline derivative **4** showed greater susceptibility to hydrolysis, potentially reflecting differences in steric and electronic properties of their amino acid moieties. Overall, the chromatograms presented in Figures S25 and S26 also suggest reduced hydrolysis of the pinocembrin–amino acid conjugates compared with their corresponding chrysin analogues. Variable rates of non-enzymatic ester hydrolysis depending on pH and the structures of both the parent drug and conjugated amino acid have also been reported for other amino acid-based prodrugs [18,19,20,21].

**Figure 3:**
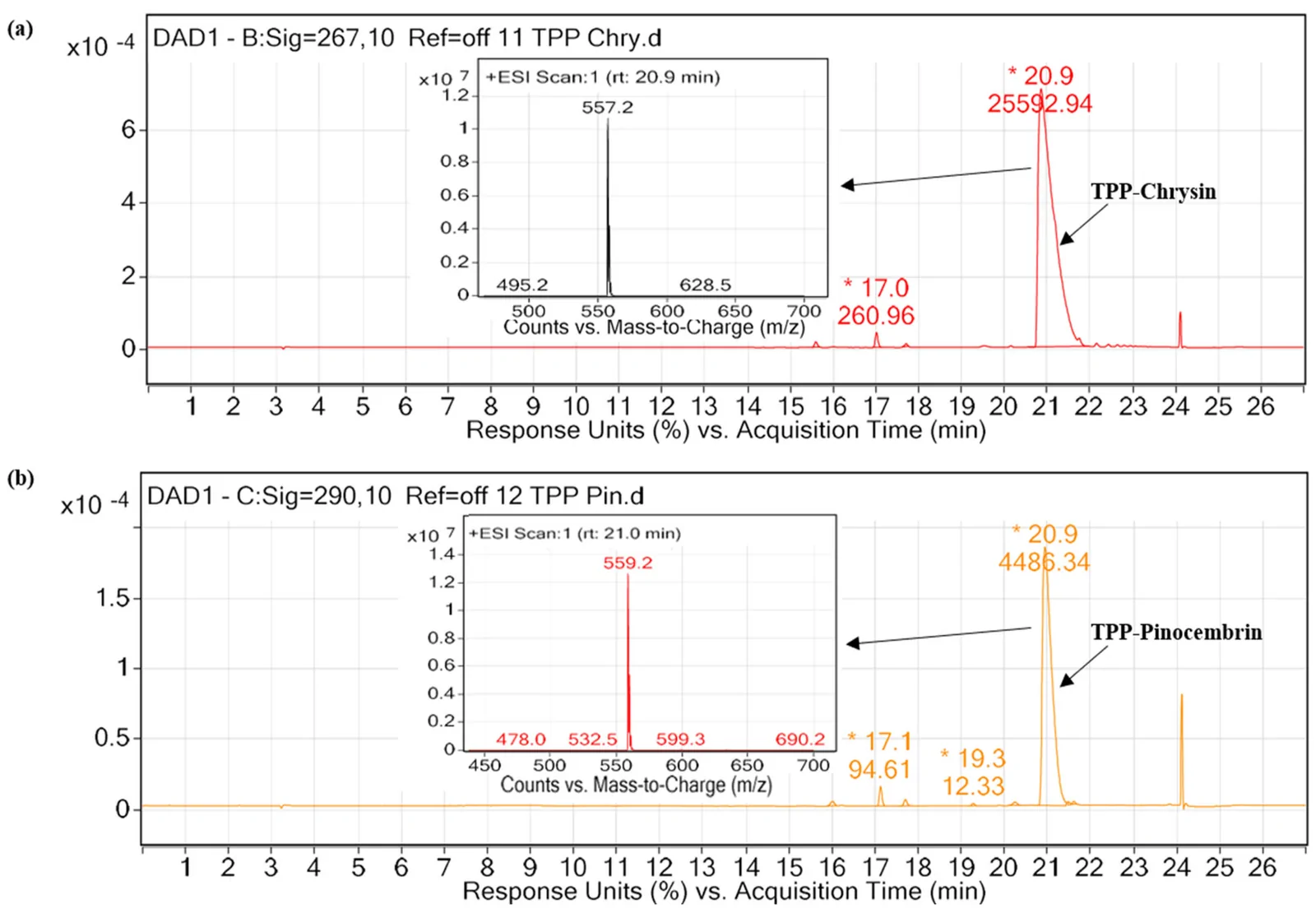
Chromatogram of TPP derivative with inset of the +ESI scan: (**a**) TPP-chrysin measured at 267 nm, (**b**) TPP-pinocembrin measured at 290 nm. (The retention time and peak area are labelled in the figures—*).

In contrast to amino acid conjugates **1**–**10**, the TPP derivatives showed no detectable decomposition under the applied chromatographic conditions. This higher stability is consistent with the presence of a stable ether linkage connecting the TPP-containing spacer to the 7-hydroxy group of the flavonoid, rather than the hydrolytically labile ester linkage present in compounds **1**–**10**. Accordingly, the chromatographic purities of compounds **11** and **12** were determined to be 98.99% and 97.67%, respectively (Figure 3).

### 2.3. Minimum Inhibitory Concentration

The MIC value was determined using the classical 2-fold serial dilution method, and the results are listed in Table 1. Chrysin and pinocembrin showed no inhibitory effect against the tested *Candida* species even at the highest tested concentration of 1024 µg/mL. On the other hand, pinocembrin showed an MIC value of 128 µg/mL against the tested *S. aureus*, while it was higher than 1024 µg/mL for *E. coli* and all tested strains in the case of chrysin. The antibacterial activity of both flavonoids and the antifungal activity of chrysin were unaffected by the AA conjugation. However, the optical density measurements (Figure S27) obtained at an equimolar concentration of AA conjugates to that of the parent flavonoid in 1024 µg/mL pointed to reduced turbidity, indirectly suggesting a possible increase in solubility. At equimolar concentrations, the AA–flavonoid conjugates introduced approximately 1.6–2.5-fold higher mass concentrations than their respective parent compounds into the culture medium. Despite this higher mass concentration, the AA–flavonoid conjugates exhibited lower optical density and a lesser amount of visible precipitation compared to the parent compounds. A lower level of precipitation of the investigated AA derivatives was observed in comparison to the respective flavonoids during the MIC assay, which can be attributed to the partial hydrolysis of the ester bonds in these compounds as indicated by the LC-MS results. Moreover, the optical density of the conjugates decreased with temperature (to 37 °C at the highest tested concentration of 1024 µg/mL), while that of free flavonoids at the same concentration was less affected by temperature. The AA conjugation of pinocembrin (compounds **7**–**10**) showed a maximum of four-fold decrease in the MIC values against *C. albicans* SC5314 and *C. krusei* DSM 6128. *C. glabrata* DSM 11226 appeared to be more resistant to the tested compounds. The hydrolysis of the AA-flavonoid ester bond in the bacterial and fungal culture medium is one of the possible reasons for the poor activity. Hydroxylation at the C5 and C7 positions and the unsubstituted B ring are structural advantages for the antibacterial activity of pinocembrin against gram-positive bacteria. Additionally, the C2=C3 double bond in flavones is beneficial for enhancing the bacterial inhibition against gram-positive bacteria [22]. The two hydroxyl groups at an ambient position in the A ring in the flavonoid can anchor the polar region of bacterial phospholipid membrane, facilitating the interaction of the unsubstituted B ring with the nonpolar region of the membrane. The planar nature of flavones is generally an advantage over the non-planar structure with a perpendicular phenyl ring of flavonones. A previous study has shown superior antibacterial activity of galangin over pinocembrin, supporting the previous notion [23]. However, chrysin deviates from this trend, potentially due to the absence of the C3-hydroxyl group. Chrysin and pinocembrin are key contributors to the synergistic antifungal activity of propolis, with the flavone being able to penetrate through the fungal membrane [8].

During the initial development of the TPP–flavonoid conjugates, the ester-linked derivative ((4-((5-hydroxy-4-oxo-2-phenyl-4H-chromen-7-yl)oxy)-4-oxobutyl)triphenylphosphonium bromide) was also investigated. However, their hydrolytic stability was not quantitatively evaluated. Given the pronounced hydrolysis observed for the amino acid–flavonoid conjugates **1**–**10**, which also contain an ester linkage at the 7-OH position, the use of an analogous ester linkage in the TPP derivatives was considered potentially problematic. In both cases, hydrolytic cleavage of the ester bond would release the corresponding flavonoid phenol, providing a structural rationale for anticipating limited hydrolytic stability. Moreover, the ester-linked TPP derivatives exhibited pronounced precipitation in aqueous media, further limiting their suitability for biological evaluation. Therefore, these derivatives were not pursued further, and the ester linkage was replaced with a chemically more stable ether linkage, leading to compounds **11** and **12**. Hence, the ester linkage for the TPP derivative was replaced with an ether bond to obtain compounds **11** and **12**. TPP derivatives synthesised with a stable ether link showed high antimicrobial potential. The MIC values of these compounds dropped significantly from 1024 µg/mL to 8 µg/mL, 16 µg/mL and 64 µg/mL against the tested *Candida* strains. Similarly, they showed high antimicrobial potency against the tested Gram-positive and Gram-negative bacteria. The observed increase in antimicrobial potency can be attributed to the stable ether bond between the cationic TPP and flavonoid moieties, which guarantees the cell permeability of both components. TPP-Br and TPP-COOH were also tested against all the strains to assess whether the antimicrobial activity of compounds **11** and **12** was primarily associated with the TPP moiety. TPP-Br showed weak antimicrobial activity against (*C. krusei* DSM 6128, *S. aureus* ATCC 29213 and *S. aureus* ATCC 25923); however, compounds **11** and **12** are bromine-free, and the bromine substituent itself could contribute to the observed activity [24]. Therefore, TPP-COOH was evaluated. Replacement of Br with a –COOH group resulted in no detectable antimicrobial activity. These findings confirm that the activity of compounds **11** and **12** is not attributable solely to the TPP moiety but instead arises from the TPP moiety facilitating the cellular availability of the conjugated flavonoid. Taking into account our research group’s interests in the subsequent stages of the study, we focused on evaluating the antifungal activity of the synthesised TPP derivatives.

**Table 1:** MIC values of amino acid and TPP derivatives of chrysin and pinocembrin in μg/mL (*n* = 3).

Compound	*C. albicans* SC5314	*C. glabrata* DSM 11226	*C. krusei* DSM 6128	*S. aureus* ATCC 29213	*S. aureus* ATCC 25923	*E. coli* ATCC 25922
**Chrysin**	>1024	>1024	>1024	>1024	>1024	>1024
**Pinocembrin**	>1024	>1024	>1024	128	128	>1024
Amphotericin B *	0.5	1	2	-	-	-
**Ampicillin**	-	-	-	0.5	0.125	2
**TPP-Br**	>256	>256	64	32	64	>256
**TPP-COOH**	>256	>256	>256	>256	>256	>256
**1**	>1024	>1024	>1024	>1024	>1024	>1024
**2**	>1024	>1024	>1024	>1024	>1024	>1024
**3**	>1024	>1024	>1024	>1024	>1024	>1024
**4**	>1024	>1024	>1024	>1024	>1024	>1024
**5**	>1024	>1024	>1024	>1024	>1024	>1024
**6**	>1024	>1024	>1024	256	256	>1024
**7**	512	512	256	128	256	>1024
**8**	256	>1024	>1024	128	256	>1024
**9**	512	512	512	256	256	>1024
**10**	256	>1024	256	128	128	>1024
**11**	8	64	8	0.5	1	64
**12**	8	64	16	0.25	0.25	16

*—based on previous studies performed in our laboratory [25].

### 2.4. Time-Kill Assay

**Figure 4:**
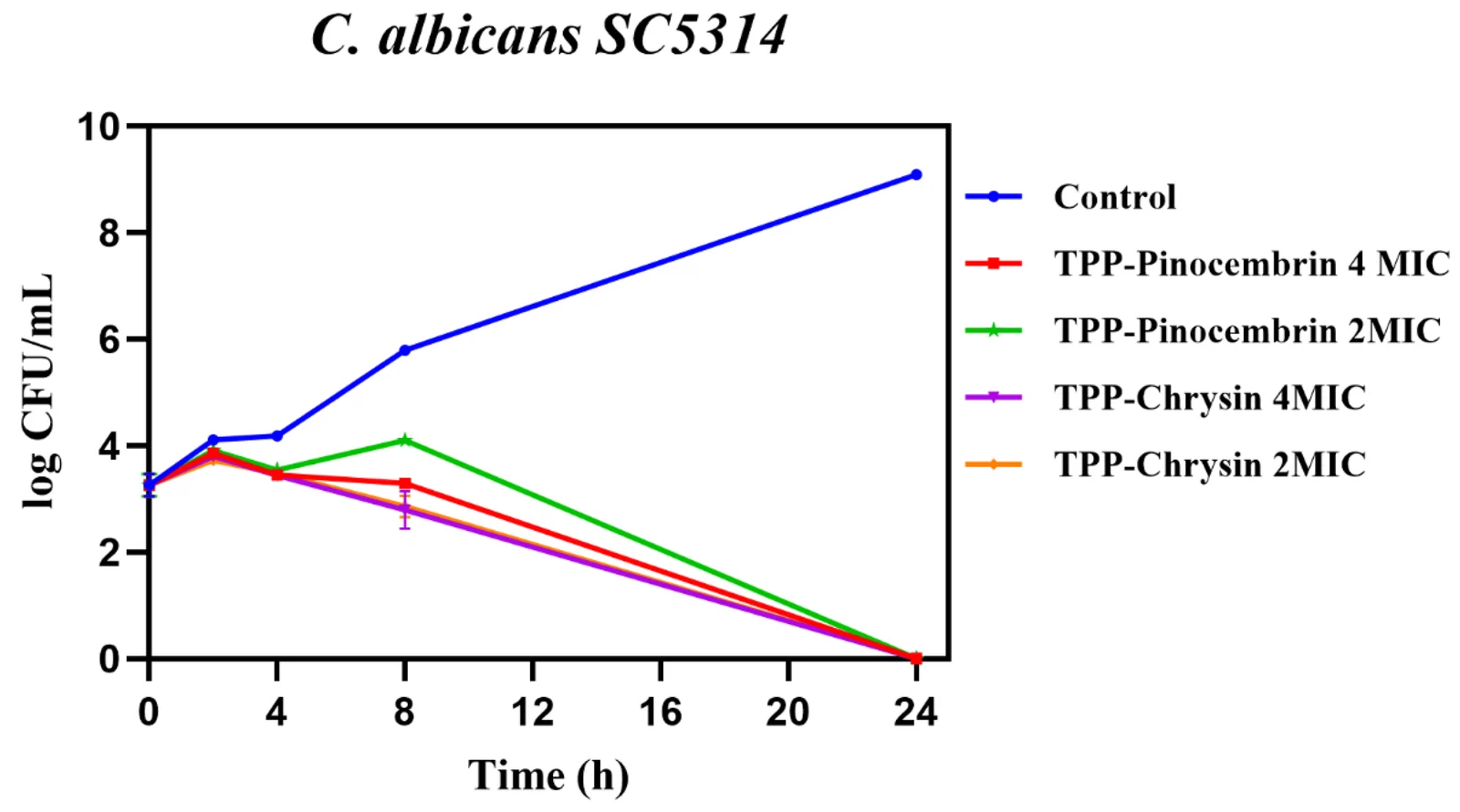
Kill time assay of TPP-Pinocembrin and TPP-Chrysin at 4 MIC and 2 MIC concentrations against *C albicans* SC5314. The control represents untreated cells. The results are shown as mean ± SD. Data points with no error bars are indicative of negligible value of SD.

The time-kill assay evaluates the kinetics of the fungicidal/bactericidal effect, enabling differentiation between the microbiostatic and microbiocidal potential of an antimicrobial agent at a given concentration over time. When *C. albicans* was treated with TPP-flavonoids, an initial increase of CFU/ml was observed at 2 h for all tested conditions. Then, it was followed by a small reduction in cell growth until 8 h (except for 2 MIC Pinocembrin). However, all the tested concentrations proved to be fungicidal against the strain over a period of 24 h (Figure 4). A clear distinction in the activity is visible for each concentration for the TPP-pinocembrin derivative in the initial hours. The difference between antifungal activity at 2 MIC and 4 MIC concentrations was relatively small in the case of TPP-Chrysin.

### 2.5. Flow Cytometry

**Figure 5:**
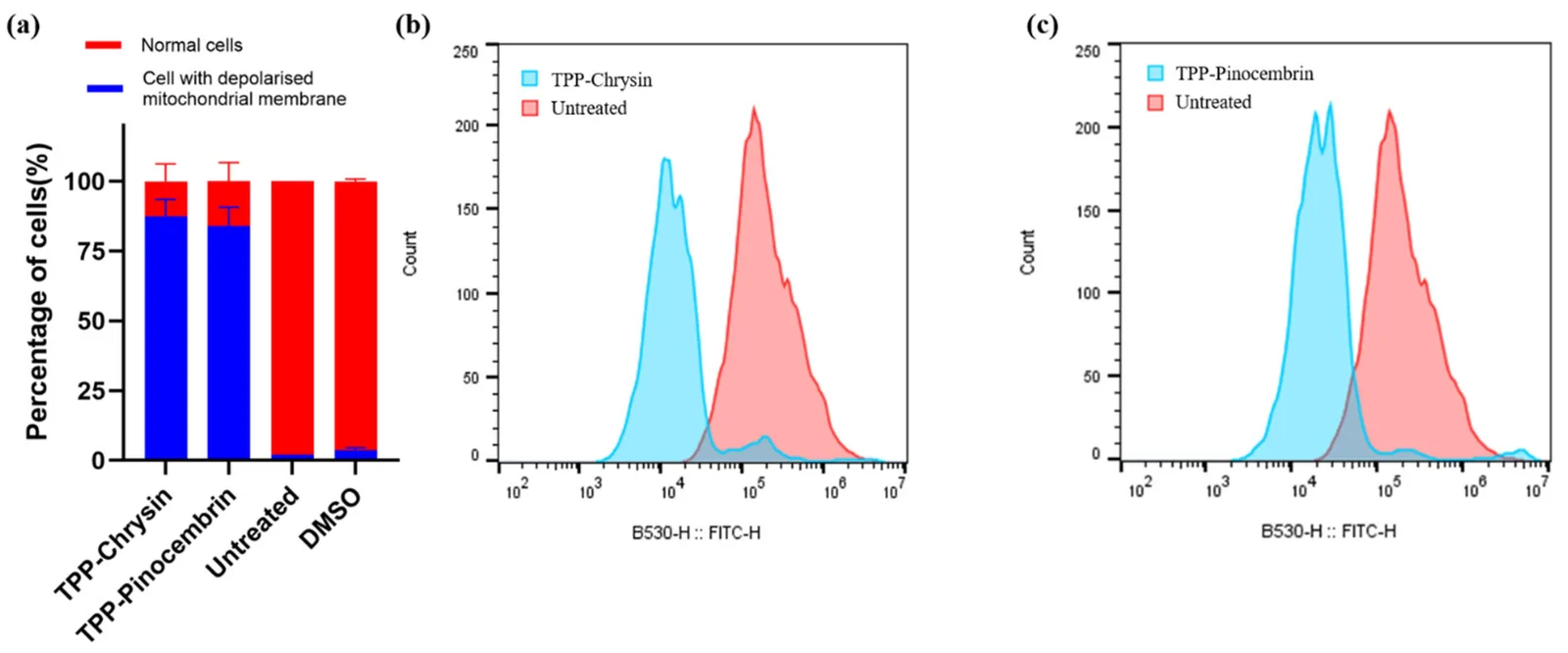
Assessment of mitochondrial membrane depolarisation in *C. albicans SC 5314* by Rh123 staining and flow cytometry. (**a**) Quantitative analysis (values are expressed as mean ± SD (*n* = 3)); representative histograms of (**b**) TPP–Chrysin and (**c**) TPP–Pinocembrin treated cells. The cells were incubated with the test compounds for 2 h.

The yeast cells were exposed to the synthesised compounds for 2 h and stained with rhodamine, 2′,7′-dichlorodihydrofluorescein diacetate or propidium iodide. Rh123 is a fluorescent dye that localises in mitochondria and can be used to assess mitochondrial membrane potential (Δψm). It enables the identification of membrane hyperpolarisation and depolarisation induced by mitochondria-targeted compounds [26]. Figure 5a. indicates that compounds **11** and **12** induced depolarisation of the mitochondrial membrane in 87.33% and 83.97% of the cell population, respectively. Figure 5b,c indicates the extent of membrane depolarisation induced by the compounds in comparison to untreated cells. The negative membrane potential of the cell membrane facilitates the lipophilic cation of TPP to penetrate the cell, and the higher negative membrane potential of mitochondria guides the incoming cationic species to accumulate in it, leading to depolarisation of the mitochondrial membrane [27]. Loss of Δψm can affect ATP production and lead to cell death in *C. albicans* [28,29,30].

**Figure 6:**
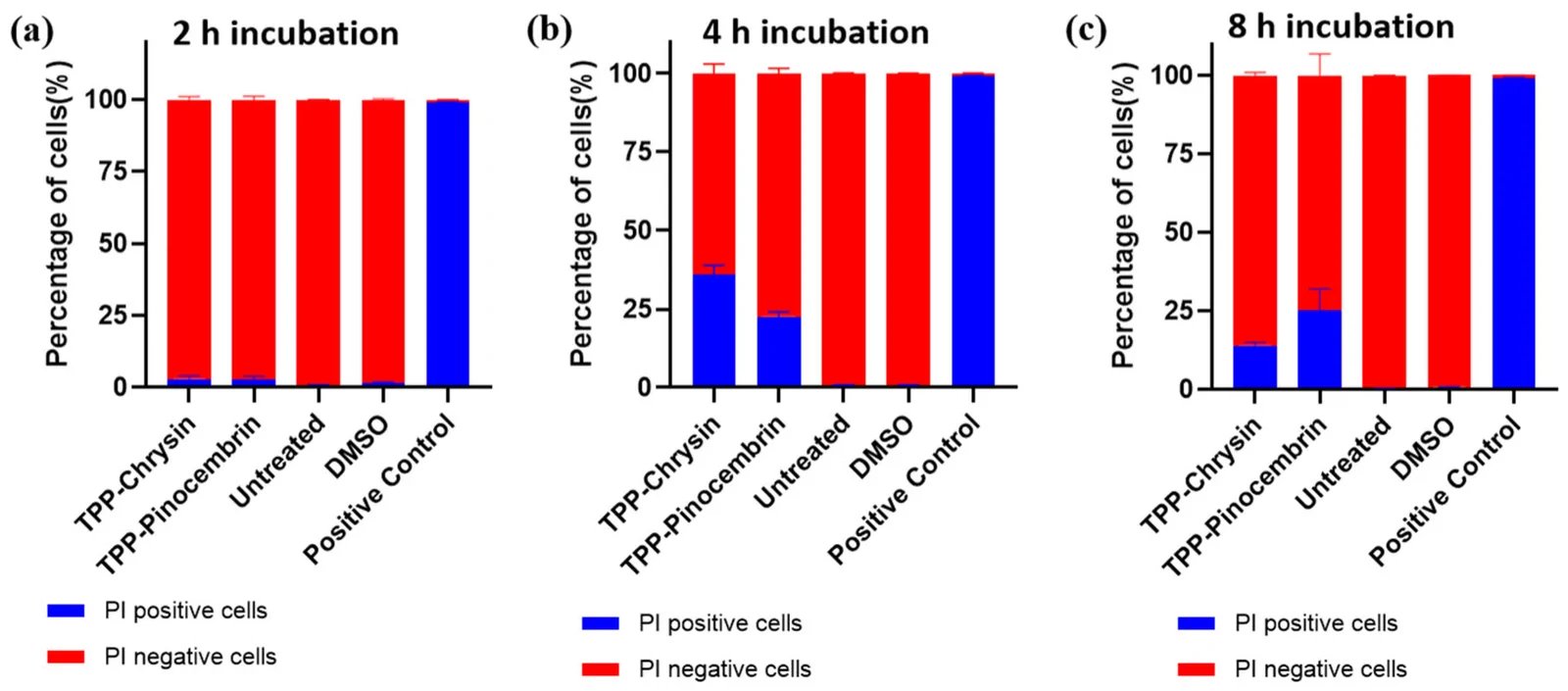
Time-dependent membrane permeabilisation of *C. albicans* SC5314 assessed by PI staining and flow cytometrtic analyses. Representative quantitative analysis after (**a**) 2 h, (**b**) 4 h, and (**c**) 8 h of treatment. EtOH acts as positive control. Values are expressed as mean ± SD (*n* = 3).

**Figure 7:**
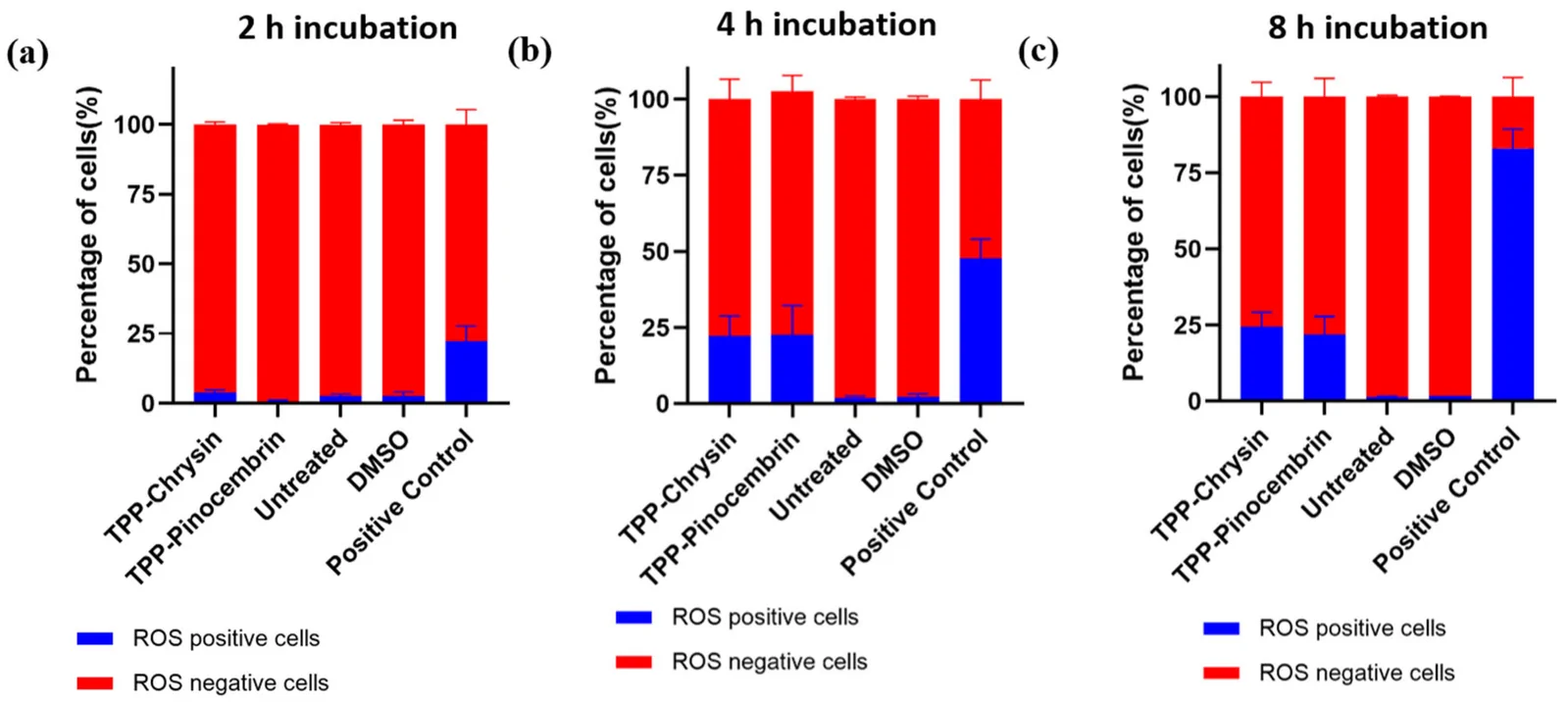
Time-dependent intracellular ROS production in *Candida albicans* SC5314 determined by H2-DCFDA staining and flow cytometry. Representative quantitative analysis after (**a**) 2 h, (**b**) 4 h, and (**c**) 8 h of treatment. 1%H_2_O_2_ is used as the positive control. Values are expressed as mean ± SD (*n* = 3).

Another study, which used mitocurcumin (TPP-curcumin) against *C. albicans,* indicated depolarisation of mitochondrial membrane, the loss of cell membrane integrity and enhanced ROS production as a mechanism of apoptosis of cells [28]. The membrane integrity and ROS-mediated cell death were also evaluated during this study using PI and H2-DCFDA fluorescent dyes. PI is a nucleic acid-binding fluorescent dye. It permeates through the damaged cell membrane and produces intense red fluorescence, often indicating cell death [25,26]. H2-DCFDA is a cell-permeable fluorescent dye indicative of oxidative stress, which emits bright green fluorescence inside the cell [31]. Time-dependent studies were performed to evaluate the impact of these compounds on membrane integrity and oxidative stress. Figure 6 and Figure 7 indicate the time-dependent PI penetration and ROS generation in the tested species over 2, 4 and 8 h. An incubation period of 2 h with compounds **11** and **12** is inadequate to induce damage to the cell membrane and oxidative stress. The incubation time, when extended to 4 h, increased the PI permeability to approximately 35% and 22.6% for compounds **11** and **12**, respectively. Further increase in incubation time had no positive impact on the PI permeability (Figure 6). T The PI permeability of TPP-Chrysin decreased at 8 h, while the time-kill assay (Figure 4) showed a further decrease in CFU/mL, indicating the continued fungicidal activity of the compound. The decrease in PI-positive cells at 8 h may therefore be associated with further progression of cell damage following prolonged exposure to the compound. Severely damaged cells may undergo complete lysis, resulting in a reduction in the number of intact PI-stained cells detected in the tested volume. A similar observation was made in the case of ROS generation. The oxidative stress induced by 1% (*v*/*v*) H_2_O_2_ increased over time, while no significant further increase in signal was observed for the tested compounds after 4 h (Figure 6). These observations imply that loss of cell membrane integrity and induced oxidative stress could act as secondary mechanisms contributing to apoptosis of cells.

Membrane depolarisation was the earliest detectable event, occurring after the shortest incubation time, suggesting that it represents the primary mechanism of action of the compound. In contrast, ROS generation and membrane permeabilisation, as indicated by PI uptake, were observed only after longer incubation periods. This temporal sequence suggests that mitochondrial membrane depolarisation may precede and contribute to the subsequent induction of oxidative stress and membrane permeabilisation.

### 2.6. Haemolytic Activity

**Figure 8:**
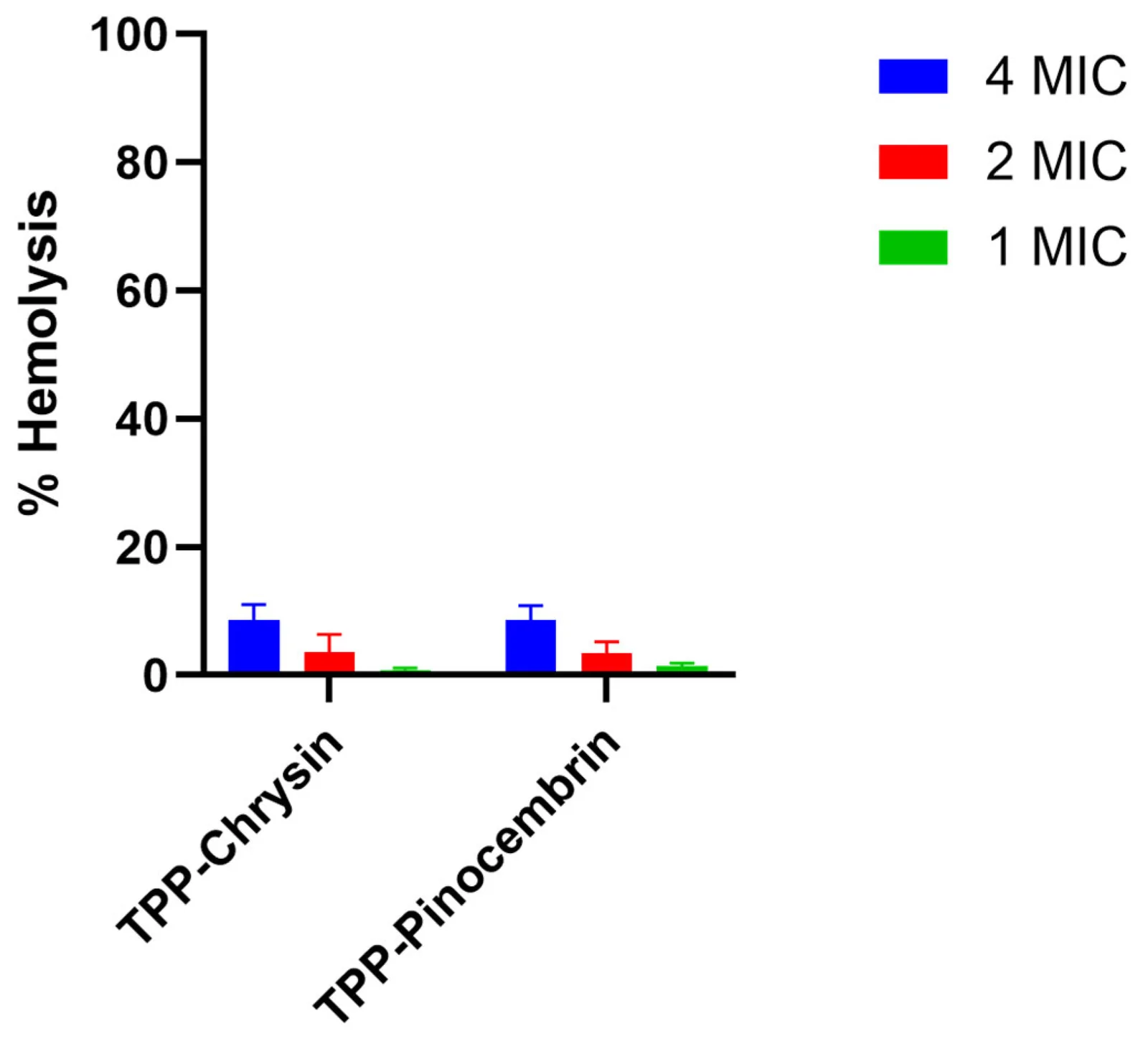
Haemolytic activity of TPP-chrysin and TPP-pinocembrin at 4 MIC, 2 MIC, and 1 MIC concentrations. Data are presented as mean ± SD (*n* = 3).

The haemolytic activity of compounds **11** and **12** was tested against defibrinated sheep blood to evaluate their biocompatibility. This assay measures the extent of red blood cell rupture and subsequent haemoglobin leakage induced by the tested compounds [32]. Three different concentrations of the compounds were tested, and the results are expressed as the percentage of haemolysishaemolysis relative to the hemolysis induced by 0.1% sodium dodecyl sulphate (SDS), as shown in Figure 8. A recent study categorised haemolytic activity into four tiers: negligible (≤10%), low (>10–25%), moderate (>25–50%), and high/severe (>50%) [33]. The haemolytic activity of compounds **11** and **12** was concentration-dependent, with the mean percentage of haemolysis ranging between 8 and 9% at the highest tested concentration. Thus, both compounds exhibited negligible haemolytic activity, indicating favourable biocompatibility under the tested conditions. This result preliminarily confirms the high selectivity (high antifungal potential and low toxicity) of both compounds, which promotes further research of their potential as antimicrobial agents and safety for host organisms (humans and animals).

## 3. Materials and Methods

### 3.1. Chemistry

#### 3.1.1. General

All solvents were used as received from commercial suppliers (Merck, Warsaw, Poland; Chemat, Gdańsk, Poland). Boc-protected lysine and serine were supplied as their dicyclohexylamine (DCHA) salts (Chemat, Gdańsk, Poland). To liberate the corresponding free amino acids, the salts were dissolved in AcOEt, washed with 10% H_3_PO_4_, dried over anhydrous MgSO_4_, and evaporated to dryness. Boc-protected glycine, leucine, and proline were used as received from commercial suppliers (Merck, Warsaw, Poland; Chemat, Gdańsk, Poland). ^1^H and ^13^C NMR spectra were recorded on a Varian Unity Plus 500 MHz spectrometer (Darmstadt, Germany) using deuterated solvents for field-frequency locking. Chemical shifts (δ) are reported in parts per million (ppm), and coupling constants (*J*) are given in hertz (Hz). Signal multiplicities are abbreviated as follows: s—singlet, d—doublet, t—triplet, q—quartet, qi—quintet, sx—sextet, sp—septet, o—octet, n—nonet, m—multiplet, and bs—broad singlet. HPLC-DAD-MS data were acquired using an Agilent 1260 Infinity II system coupled to an Agilent 6470 Triple Quadrupole LC/MS system (Agilent Technologies, Santa Clara, CA, USA). HRMS spectra were recorded using an Agilent 6540 UHD QTOF-MS system (Agilent Technologies, Santa Clara, CA, USA). Column chromatography was performed on silica gel 0.040–0.063 mm (Chemat, Gdańsk, Poland) using the indicated solvent systems.

#### 3.1.2. Synthesis

***Amino acid:chrysin conjugates—general procedure***. Chrysin (1.96 mmol), Boc-protected amino acid (2.35 mmol), and DMAP (2.35 mmol) were dissolved in dry DCM in an ice bath. Then EDCI (3.53 mmol) was added in one portion, and the resulting mixture was stirred overnight at room temperature. The reaction mixture was washed with 1 M HCl, brine and saturated NaHCO_3_ solutions. The organic layer was dried over anhydrous MgSO_4_, the desiccant was filtered off, and the filtrate was removed under reduced pressure. The residue dissolved was recrystallised from *i*PrOH to obtain the intermediate compound. The obtained Boc-protected intermediates were dissolved in a 3/1 (*v*/*v*) DCM: TFA mixture and stirred for 30 min at room temperature, and then the solvents were evaporated under reduced pressure. The desired products precipitated as TFA salts from the crude concentrated mixture upon the addition of Et_2_O.

*Gly:chrysin* (**1**). Coupling of chrysin (500 mg, 1.96 mmol) with Boc-Gly-OH (412 mg, 2.35 mmol), using EDCI (728 mg, 3.53 mmol) and DMAP (278 mg, 2.35 mmol), followed by Boc deprotection, afforded conjugate **1** (273.5 mg, 0.643 mmol, 32.8%) as a pale yellow solid. HRMS (ESI): calcd. for C_17_H_14_NO_5_^+^ *m*/*z*: 312.0866, found: 312.0882. ^1^H NMR (500 MHz, DMSO-*d_6_*) δ [ppm]: 12.89 (s, 1H), 8.62 (s, 3H), 8.10 (d, *J* = 7.2 Hz, 2H), 7.60 (dt, *J* = 25.4, 7.2 Hz, 3H), 7.15 (s, 1H), 7.12 (d, *J* = 2.0 Hz, 1H), 6.69 (d, *J* = 2.0 Hz, 1H), 4.16 (s, 2H). ^13^C NMR (126 MHz, DMSO-*d_6_*) δ [ppm] 183.01, 166.52, 164.72, 161.34, 157.86, 155.27, 132.90, 130.70, 129.62, 127.06, 109.10, 106.28, 105.56, 105.37, 40.62.

*Leu:chrysin* (**2**). Coupling of chrysin (500 mg, 1.96 mmol) with Boc-Leu-OH (586 mg, 2.35 mmol), using EDCI (728 mg, 3.53 mmol) and DMAP (278 mg, 2.35 mmol), followed by Boc deprotection, afforded conjugate **2** (313.5mg, 0.6517 mmol, 33.25%) as a pale yellow solid. HRMS (ESI): calcd. for C_21_H_22_NO_5_^+^ *m*/*z*: 368.1492, found: 368.1517. ^1^H NMR (500 MHz, DMSO-*d_6_*) δ [ppm]: 12.91 (s, 1H), 8.75 (s, 3H), 8.11 (d, *J* = 7.6 Hz, 2H), 7.66–7.55 (m, 3H), 7.18 (d, *J* = 2.2 Hz, 1H), 7.17 (s, 1H), 6.72 (d, *J* = 2.2 Hz, 1H), 4.31 (t, *J* = 6.8 Hz, 1H), 1.87 (td, *J* = 14.4, 6.6 Hz, 2H), 1.78 (q, *J* = 6.3 Hz, 1H), 0.97 (t, *J* = 5.4 Hz, 6H). ^13^C NMR (126 MHz, DMSO-*d_6_*) δ [ppm] 183.05, 168.59, 164.73, 161.37, 156.77, 155.20, 132.93, 130.71, 129.63, 127.07, 109.19, 106.28, 105.39, 101.87, 51.23, 39.40, 24.33, 22.53, 22.47.

*Lys:chrysin* (**3**). Coupling of chrysin (590 mg, 2.323 mmol) with Boc-Lys(Boc)-OH (966 mg, 2.788 mmol), using EDCI (801.68 mg, 4.18 mmol) and DMAP (340.60 mg, 2.788 mmol), followed by Boc deprotection, afforded conjugate **3** (610 mg, 1.018 mmol, 43.88%) as a reddish brown solid. HRMS (ESI): calcd. for C_21_H_24_N_2_O_5_^2+^ *m*/*z*: 384.1674, found: 383.1617. ^1^H NMR (500 MHz, DMSO-*d_6_*) δ [ppm]: 12.94 (s, 1H), 8.66 (s, 3H), 8.19–8.09 (m, 2H), 7.75 (s, 3H), 7.67–7.57 (m, 3H), 7.21 (s, 1H), 7.20 (d, *J* = 1.9 Hz, 1H), 6.75 (d, *J* = 1.9 Hz, 1H), 4.35 (s, 1H), 2.82 (t, *J* = 7.1 Hz, 2H), 2.04–1.87 (m, 2H), 1.61–1.45 (m, 4H). ^13^C NMR (126 MHz, DMSO-*d_6_*) δ [ppm] 183.09, 168.15, 164.80, 161.40, 156.79, 155.19, 132.97, 130.76, 129.66, 127.10, 109.23, 106.35, 105.60, 105.51, 52.47, 40.72, 29.87, 28.87, 21.80.

*Pro:chrysin* (**4**). Coupling of chrysin (500 mg, 1.96 mmol) with Boc-Pro-OH (506.28 mg, 2.35 mmol), using EDCI (728 mg, 3.53 mmol) and DMAP (278 mg, 2.35 mmol), followed by Boc deprotection, afforded conjugate **4** (349.5 mg, 0.7516 mmol, 38.34%) as a yellow solid. HRMS (ESI): calcd. for C_20_H_18_NO_5_^+^ *m*/*z*: 352.1179, found: 352.1205. ^1^H NMR (500 MHz, DMSO-*d_6_*) δ [ppm]: 12.89 (s, 1H), 8.09 (d, *J* = 7.2 Hz, 2H), 7.74–7.44 (m, 5H), 7.22 (d, *J* = 2.0 Hz, 1H), 7.16 (s, 1H), 6.78 (d, *J* = 2.0 Hz, 1H), 4.74 (dd, *J* = 8.8, 7.4 Hz, 1H), 3.40–3.31 (m, 2H), 2.45–2.37 (m, 1H), 2.35–2.25 (m, 1H), 2.00 (td, *J* = 7.3, 4.0 Hz, 2H). ^13^C NMR (126 MHz, DMSO-*d_6_*) δ [ppm] 183.08, 167.48, 164.76, 161.33, 156.74, 155.28, 132.94, 130.76, 129.67, 127.07, 109.20, 106.33, 105.56, 101.97, 59.19, 46.20, 28.14, 23.63.

*Ser:chrysin* (**5**). Coupling of chrysin (710 mg, 2.796 mmol) with Boc-Ser(*t*Bu)-OH (688.5 mg, 3.355 mmol), using EDCI (964.78 mg, 5.0328 mmol) and DMAP (278 mg, 2.35 mmol), followed by Boc deprotection, afforded conjugate **5** (496.5 mg, 0.971 mmol, 39.03%) as a yellow solid. HRMS (ESI): calcd. for C_18_H_16_NO_6_^+^ *m*/*z*: 342.0972, found: 342.0989. ^1^H NMR (500 MHz, DMSO-*d_6_*) δ [ppm]: 12.92 (s, 1H), 8.64 (s, 3H), 8.12 (d, *J* = 7.1 Hz, 2H), 7.64–7.57 (m, 3H), 7.19 (s, 1H), 7.15 (d, *J* = 2.1 Hz, 1H), 6.71 (d, *J* = 2.1 Hz, 1H), 4.95 (s, 1H), 4.52 (s, 1H), 4.09 (dd, *J* = 12.0, 3.8 Hz, 1H), 3.89 (dd, *J* = 11.9, 3.2 Hz, 1H). ^13^C NMR (126 MHz, DMSO-*d_6_*) δ [ppm] 183.07, 167.00, 164.80, 161.37, 156.80, 155.32, 132.94, 130.75, 129.65, 127.11, 109.19, 106.34, 105.42, 101.86, 59.97, 55.11.

***Amino acid:pinocembrin conjugates—general procedure***. Pinocembrin (0.98 mmol), Boc-protected amino acid (1.18 mmol), and DMAP (1.18 mmol) were dissolved in dry DCM in an ice bath. Then EDCI (1.76 mmol) was added in one portion, and the resulting mixture was stirred overnight at room temperature. The reaction mixture was washed with 1 M HCl, brine and saturated NaHCO_3_ solutions. The organic layer was dried over anhydrous MgSO_4_, the desiccant was filtered off, and the filtrate was removed under reduced pressure. The residue was subjected to column chromatography (hexane/AcOEt, 8/2, *v*/*v*) to obtain the intermediate compound. The obtained Boc-protected intermediates were dissolved in a 3/1 (*v*/*v*) DCM/TFA mixture and stirred for 30 min at room temperature, and then the solvents were evaporated under reduced pressure. The desired products precipitated as TFA salts from the crude concentrated mixture upon the addition of Et_2_O.

*Gly:pinocembrin* (**6**). Coupling of pinocembrin (250 mg, 0.98 mmol) with Boc-Gly-OH (206 mg, 1.18 mmol), using EDCI (368 mg, 1.76 mmol) and DMAP (144 mg, 1.76 mmol), followed by column chromatography and Boc deprotection, afforded conjugate **6** (158 mg, 0.37 mmol, 37.75%) as a white solid. HRMS (ESI): calcd. for C_17_H_16_NO_5_^+^ *m*/*z*: 314.1023, found: 314.1035. ^1^H NMR (500 MHz, DMSO-*d_6_*) δ [ppm]: 11.97 (s, 1H), 8.49 (s, 3H), 7.54 (d, *J* = 9.8 Hz, 2H), 7.49–7.36 (m, 3H), 6.43 (d, *J* = 2.2 Hz, 1H), 6.40 (d, *J* = 2.1 Hz, 1H), 5.76 (dd, *J* = 13.1, 2.9 Hz, 1H), 4.12 (s, 2H), 3.47 (dd, *J* = 17.2, 13.1 Hz, 1H), 2.92 (dd, *J* = 17.1, 3.0 Hz, 1H). ^13^C NMR (126 MHz, DMSO-*d_6_*) δ [ppm] 198.25, 166.36, 162.68, 162.64, 157.31, 138.62, 129.25, 129.10, 127.17, 106.75, 102.90, 101.81, 79.28, 42.79, 40.57.

*Leu:pinocembrin* (**7**). Coupling of pinocembrin (250 mg, 0.98 mmol) with Boc-Leu-OH (293 mg, 1.18 mmol), using EDCI (368 mg, 1.76 mmol) and DMAP (144 mg, 1.76 mmol), followed by column chromatography and Boc deprotection, afforded conjugate **7** (106.9mg, 0.2213 mmol, 22.58%) as a white solid. HRMS (ESI): calcd. for C_21_H_24_NO_5_^+^ *m*/*z*: 370.1649, found: 370.1664. ^1^H NMR (500 MHz, DMSO-*d_6_*) δ [ppm]: 11.97 (s, 1H), 8.69 (s, 3H), 7.54 (d, *J* = 9.0 Hz, 2H), 7.50–7.33 (m, 3H), 6.46 (d, *J* = 2.2 Hz, 1H), 6.43 (d, *J* = 2.2 Hz, 1H), 5.76 (d, *J* = 11.6 Hz, 1H), 4.27 (t, *J* = 6.9 Hz, 1H), 3.54–3.41 (m, 1H), 2.93 (dd, *J* = 17.1, 3.1 Hz, 1H), 1.84 (dp, *J* = 13.3, 6.7 Hz, 2H), 1.78–1.68 (m, 1H), 0.97–0.93 (m, 6H). ^13^C NMR (126 MHz, DMSO-*d_6_*) δ [ppm] 198.31, 168.47, 162.72, 162.67, 157.20, 138.63, 129.26, 129.10, 127.17, 106.84, 102.88, 101.83, 79.27, 51.20, 42.81, 24.31, 22.54, 22.44.

*Lys:pinocembrin* (**8**). Coupling of pinocembrin (308.37 mg, 1.2046 mmol) with Boc-Lys(Boc)-OH (500.5 mg, 1.4455 mmol), using EDCI (415 mg, 2.1683 mmol) and DMAP (176 mg, 1.4455 mmol), followed by column chromatography and Boc deprotection, afforded conjugate **8** (146.3mg, 0.2390 mmol, 19.92%) as a reddish-brown solid. HRMS (ESI): calcd. for C_21_H_26_N_2_O_5_^2+^ *m*/*z*: 386.1831, found: 385.1776. ^1^H NMR (500 MHz, DMSO-*d_6_*) δ [ppm]: 11.98 (s, 1H), 8.70 (s, 3H), 7.81 (s, 3H), 7.54 (d, *J* = 7.3 Hz, 2H), 7.44 (dt, *J* = 14.2, 7.0 Hz, 3H), 6.49 (d, *J* = 2.3 Hz, 1H), 6.45 (s, 1H), 5.76 (d, *J* = 14.0 Hz, 1H), 4.30 (s, 1H), 3.47 (ddd, *J* = 16.1, 12.9, 2.8 Hz, 1H), 2.93 (dd, *J* = 17.3, 3.1 Hz, 1H), 2.86–2.70 (m, 2H), 2.06–1.80 (m, 2H), 1.68–1.38 (m, 4H). ^13^C NMR (126 MHz, DMSO-*d_6_*) δ [ppm] 198.34, 168.03, 163.94, 162.65, 157.20, 139.16, 129.28, 129.02, 127.17, 106.84, 103.02, 102.22, 79.30, 52.42, 42.80, 40.73, 29.85, 28.88, 21.75.

*Pro:pinocembrin* (**9**). Coupling of pinocembrin (250 mg, 0.98 mmol) with Boc-Pro-OH (206.14mg, 1.18 mmol), using EDCI (368 mg, 1.76 mmol) and DMAP (144 mg, 1.76 mmol), followed by column chromatography and Boc deprotection, afforded conjugate **9** (159 mg, 0.3404 mmol, 34.73%) as a white solid. HRMS (ESI): calcd. for C_20_H_20_NO_5_^+^ *m*/*z*: 354.1336, found: 354.1366. ^1^H NMR (500 MHz, DMSO-*d_6_*) δ [ppm]: 11.97 (s, 1H), 9.37 (s, 2H), 7.54 (d, *J* = 9.0 Hz, 2H), 7.49–7.32 (m, 3H), 6.53 (d, *J* = 2.2 Hz, 1H), 6.49 (d, *J* = 2.2 Hz, 1H), 5.76 (dd, *J* = 12.9, 3.1 Hz, 1H), 4.68 (t, *J* = 8.1 Hz, 1H), 3.46 (dd, *J* = 17.2, 12.9 Hz, 1H), 3.36–3.21 (m, 2H), 2.93 (dd, *J* = 17.2, 3.2 Hz, 1H), 2.44–2.18 (m, 2H), 1.97 (pt, *J* = 7.3, 4.0 Hz, 2H). ^13^C NMR (126 MHz, DMSO-*d_6_*) δ [ppm] 198.33, 167.38, 162.67, 162.60, 157.32, 138.65, 129.24, 129.11, 127.13, 106.83, 103.06, 101.99, 79.25, 59.18, 46.21, 42.79, 28.13, 23.60.

*Ser:pinocembrin* (**10**). Coupling of pinocembrin (504.10 mg, 1.96 mmol) with Boc-Ser(*t*Bu)-OH (484.9 mg, 2.363 mmol), using EDCI (543.58 mg, 2.8356 mmol) and DMAP (288 mg, 2.363 mmol), followed by column chromatography and Boc deprotection, afforded conjugate **10** (63.2 mg, 0.138 mmol, 7%) as a white solid. HRMS (ESI): calcd. for C_18_H_18_NO_6_^+^ *m*/*z*: 344.1129, found: 344.1142. ^1^H NMR (500 MHz, DMSO-*d_6_*) δ [ppm]: 11.97 (s, 1H), 8.63 (s, 3H), 7.54 (d, *J* = 7.4 Hz, 2H), 7.43 (dt, *J* = 21.4, 7.0 Hz, 3H), 6.44 (s, 1H), 6.41 (d, *J* = 2.3 Hz, 1H), 5.84 (s, 1H), 5.76 (d, *J* = 16.3 Hz, 1H), 4.47 (t, *J* = 3.5 Hz, 1H), 4.05 (d, *J* = 16.0 Hz, 1H), 3.87 (d, *J* = 11.8 Hz, 1H), 3.48 (dd, *J* = 16.1, 3.4 Hz, 1H), 2.93 (dd, *J* = 17.3, 3.2 Hz, 1H). ^13^C NMR (126 MHz, DMSO-*d_6_*) δ [ppm] 198.30, 166.88, 162.69, 162.64, 157.39, 138.62, 129.26, 129.11, 127.16, 106.81, 102.95, 101.89, 79.28, 59.95, 55.07, 42.79.

*TPP^+^:chysin* (**11**). Chrysin (747 mg, 2.94 mmol) was treated with (3-bromopropyl)triphenylphosphonium bromide (1 g, 2.16 mmol) and K_2_CO_3_ (542 mg, 3.92 mmol) in anhydrous DMF. The reaction was stirred overnight at 75 °C, then cooled to room temperature and diluted with DCM. The mixture was washed with 10% NaBr solution, the organic layer was dried over anhydrous MgSO_4,_ the desiccant was filtered off and the filtrate was concentrated under reduced pressure. The residue was recrystallised from MeOH/Et_2_O mixture, yielding conjugate **11** (652 mg, 1.0227 mmol, 34.78%) as a yellow solid.

HRMS (ESI): calcd. for C_36_H_30_O_4_P^+^ *m*/*z*: 557.1876, found: 557.1685. ^1^H NMR (500 MHz, DMSO-*d_6_*) δ [ppm]: 12.80 (s, 1H), 8.09 (d, *J* = 6.7 Hz, 2H), 7.91–7.87 (m, 3H), 7.86–7.81 (m, 6H), 7.76 (dq, *J* = 7.8, 3.6 Hz, 6H), 7.65–7.54 (m, 3H), 7.05 (s, 1H), 6.81 (d, *J* = 2.2 Hz, 1H), 6.40 (d, *J* = 2.2 Hz, 1H), 4.24 (t, *J* = 6.0 Hz, 2H), 3.80–3.70 (m, 2H), 2.06–1.96 (m, 2H). ^13^C NMR (126 MHz, DMSO-*d_6_*) δ [ppm] 182.54, 164.53, 163.94, 161.58, 157.72, 135.47, 135.45, 134.17, 134.09, 132.69, 130.98, 130.80, 130.70, 129.64, 126.90, 119.07, 118.39, 105.84, 105.52, 99.11, 93.85, 68.04, 67.89, 22.31, 18.24, 17.81.

*TPP^+^:pinocembrin* (**12**). Pinocembrin (250 mg, 0.98 mmol) was treated with (3-bromopropyl)triphenylphosphonium bromide (499 mg, 1.07 mmol) and K_2_CO_3_ (270 mg, 1.95 mmol) in anhydrous DMF. The reaction was stirred overnight at 75 °C, then cooled to room temperature and diluted with DCM. The mixture was washed with 10% NaBr solution, the organic layer was dried over anhydrous MgSO_4,_ the desiccant was filtered off and the filtrate was concentrated under reduced pressure. The residue was purified by column chromatography using gradient mobile phases: CHCl_3_/MeOH, 15/1, 10/1, and 1/1, *v*/*v* as a pale yellow solid, yielding conjugate **12** (202.4 mg, 0.3165 mmol, 32.29%) as a pale yellow solid.

HRMS (ESI): calcd. for C_36_H_32_O_4_P^+^ *m*/*z*: 559.2033, found: 559.2053. ^1^H NMR (500 MHz, DMSO-*d_6_*) δ [ppm]: 12.09 (s, 1H), 7.94–7.88 (m, 3H), 7.83 (dd, *J* = 12.5, 7.0 Hz, 6H), 7.77 (dt, *J* = 8.1, 4.1 Hz, 6H), 7.53 (d, *J* = 6.9 Hz, 2H), 7.43 (dt, *J* = 15.7, 7.8 Hz, 3H), 6.15 (d, *J* = 2.3 Hz, 1H), 6.10 (d, *J* = 2.3 Hz, 1H), 5.64 (dd, *J* = 12.5, 3.2 Hz, 1H), 4.17 (t, *J* = 6.2 Hz, 2H), 3.77–3.66 (m, 2H), 3.32 (d, *J* = 12.6 Hz, 1H), 2.85 (dd, *J* = 17.2, 3.1 Hz, 1H), 1.97 (t, *J* = 6.3 Hz, 2H). ^13^C NMR (126 MHz, DMSO-*d_6_*) δ [ppm] 197.11, 166.64, 163.60, 163.07, 138.94, 135.46, 134.16, 134.08, 130.80, 130.70, 129.14, 129.06, 127.14, 119.06, 118.37, 103.24, 95.77, 94.83, 79.05, 67.84, 67.70, 55.42, 42.57, 25.96, 22.28, 18.17, 17.75.

### 3.2. LC-MS

LC–MS analysis was performed using an Agilent 1290 Infinity II liquid chromatography system equipped with a binary pump (G7112B), autosampler (G7129A), diode array detector (DAD; G1315B), and coupled to an Agilent 6470 Triple Quadrupole (QQQ) mass spectrometer fitted with an Agilent Jet Stream electrospray ionisation (AJS-ESI) source. Chromatographic separation was achieved using an Agilent ZORBAX Eclipse XDB-C18 analytical column (150 × 4.6 mm i.d., 5 μm particle size). All compounds were dissolved in acetonitrile and filtered to remove any residual particles. The mobile phase consisted of solvent A -water containing 10 mM ammonium formate and 0.1% formic acid, and solvent B- acetonitrile. Separation was performed at a flow rate of 1.0 mL/min using the following gradient program: 95% A (0–5 min), a linear gradient to 50% A (11 min), and 100% B (20–21 min), followed by re-equilibration to 95% A until the end of the run (27 min). The injection volume was 5 μL. Mass spectrometric detection was carried out in both positive and negative electrospray ionisation modes over an m/z range of 50–700. The capillary voltage was set at ±3.5 kV, the charging voltage at 500 V, and the fragmentor voltage at 200 V. Source conditions were as follows: drying gas temperature 300 °C, drying gas flow 5 L min^−1^, sheath gas temperature 250 °C, sheath gas flow 11 L min^−1^, and nebuliser pressure 45 psi. Data acquisition was performed in full-scan mode with a scan time of 200 ms. Chromatograms were simultaneously monitored using the diode array detector at 254, 267, 280, 290, and 325 nm. UV spectra were acquired over a wavelength range of 190–400 nm with a spectral resolution of 2 nm and a slit width of 4 nm.

### 3.3. Minimum Inhibitory Concentration

The antifungal and antibacterial activities of the synthesised samples were tested against *C. albicans* SC5314, *C. glabrata* DSM 11226 (also known as *Nakaseomyces glabratus* DSM 11226), *C. krusei* DSM 6128 (also known as *Pichia kudriavzevii* DSM 6128), *S. aureus* ATCC 29213, *S. aureus* ATCC 25923 and *E. coli* ATCC 25922 using the two-fold serial dilution method with minor modifications. The procedure was previously reported [7]. The concentration of stock solution for compounds **1**–**10** was 40.960 mg/mL and 11–12 was 10.24 mg/mL to obtain a maximum final concentration of 1024 μg/mL and 256 μg/mL in the 96-well microtiter plate. 3-(bromopropyl)triphenylphosphonium bromide (TPP-Br) and (3-Carboxypropyl)triphenylphosphonium bromide (TPP-COOH) were used as a control to assess the activity of the TPP moiety. Amphotericin B and ampicillin were used against the fungal and bacterial strains, respectively. The results were confirmed by visual evaluation, optical density measurement and resazurin test. All the experiments were performed in triplicates.

### 3.4. Optical Density Measurement

The absorbance of compounds **1**–**10** in the bacterial and fungal growth media was evaluated at 531 nm on a Victor3 microplate reader (Perkin Elmer, Waltham, MA, USA) to assess their potential contribution to optical density measurements. Each compound was prepared in the respective growth medium at a molar concentration equivalent to that corresponding to 1024 µg/mL of its respective parent flavonoid. The absorbance was measured at 531 nm under the same conditions used for the antimicrobial assays.

### 3.5. Time-Kill Assay

A time-kill assay was done for compounds **11** and **12**, following a previously reported procedure [34]. The compounds were tested against *C. albicans* SC5314 at 4 MIC and 2 MIC concentrations. An overnight culture of cells was suspended in sterile PBS. The optical density of these suspensions was adjusted to 0.1 at 660 nm. This was further diluted in RPMI medium and divided into 5 equal parts. Calculated volumes of stock solution of compounds **11** and **12** were added to obtain a final concentration of 2 MIC and 4 MIC. A control with no antimicrobial agent was also used. All experiments were done in triplicate. 50 μL of each part with appropriate dilutions were spread on a SAB agar plate at time points of 0, 2, 4, 8 and 24 h. These plates were incubated overnight, and the number of colonies was counted.

### 3.6. Flow Cytometric Analysis

#### 3.6.1. General Experimental Procedure

Flow cytometric analysis of the potential molecular mechanisms of antifungal activity of synthesised compounds followed a previous procedure with minor modifications [35]. An overnight culture of *C. albicans* SC5314 grown in YPD medium was used for all flow cytometric experiments. The culture was centrifuged at 10,000 rpm for 5 min, and the supernatant was discarded. The residual pellet of cells was resuspended in PBS buffer or RPMI 1640 medium supplemented with 2% glucose to obtain a cell suspension equivalent to a 3 McFarland standard. For experiments evaluating cell membrane integrity and mitochondrial membrane potential, cell suspensions were prepared in RPMI 1640 medium containing 2% glucose. For intracellular reactive oxygen species (ROS) measurements, cell suspensions were prepared in PBS. The final concentration of the tested samples, compounds **11** and **12**, in the analysis was 4 MIC. Untreated cell suspensions and suspensions containing DMSO at concentrations equivalent to those in the corresponding treated samples were used as controls. All suspensions (treated and untreated) were incubated for 2–8 h at 37 °C with orbital shaking at 180 rpm.

Propidium iodide (PI), 2′,7′-dichlorodihydrofluorescein diacetate (H_2_DCFDA) and rhodamine 123 (Rh123) used in the experiment were purchased from Thermo Fisher Scientific (Waltham, MA, USA). Flow cytometric analyses were performed using an Agilent NovoCyte 2060R flow cytometer equipped with a NovoSampler Pro NS200 (Agilent Technologies Inc., Santa Clara, CA, USA). Samples were analysed in sterile 5 mL (12 × 75 mm) polystyrene round-bottom tubes (Corning Inc., Corning, NY, USA).

#### 3.6.2. Assessment of Cell Membrane Integrity

Following a 2 h, 4 h and 8 h exposure to TPP derivatives in RPMI medium, cell suspensions were centrifuged at 10,000 rpm for 5 min and washed once with 0.85% NaCl solution. PI was added to a final concentration of 3 µM, and the samples were incubated for 30 min at 37 °C with shaking at 180 rpm in the dark. After staining, cells were collected by centrifugation, resuspended in 0.85% NaCl solution, and immediately analysed by flow cytometry. PI fluorescence was used as an indicator of membrane permeabilisation.

#### 3.6.3. Determination of Intracellular Reactive Oxygen Species

Intracellular ROS production was determined using H2DCFDA. Cell suspensions prepared in PBS were incubated with 5 µM H2DCFDA for 30 min at 37 °C to allow intracellular dye loading. Following incubation, the cells were centrifuged at 10,000 rpm for 5 min, the supernatant was discarded, and the pellet was resuspended in RPMI medium. TPP-derivatives were then introduced to the suspension to attain a final concentration of 4 MIC. Cells treated with hydrogen peroxide, giving a final concentration of 1% (*v*/*v*) served as the positive control. After incubation for 2 h, 4 h and 8 h at 37 °C with shaking at 180 rpm, the cells were centrifuged, washed, resuspended in 0.85% NaCl solution, and analysed by flow cytometry.

#### 3.6.4. Assessment of Mitochondrial Membrane Potential

Changes in mitochondrial membrane potential were evaluated using rhodamine 123 (Rh123). Following a 2-h exposure to compounds **11** and **12** in RPMI media, cells were centrifuged and washed with citrate buffer (50 mM sodium citrate supplemented with 2% glucose and adjusted to pH 5.0). Rh123 was added to a final concentration of 25 µM, and the samples were incubated for 30 min at 37 °C in the dark. After staining, cells were centrifuged, resuspended in 0.85% NaCl solution, and analysed by flow cytometry.

### 3.7. Haemolytic Activity

The haemolytic activity of compounds was evaluated using defibrinated sheep blood collected from certified sources. The assay followed a previously mentioned procedure with minor modifications [36]. The red blood cells (RBC) were separated from whole blood by centrifuging at room temperature for 5 min at 2500 rpm. The RBC that settles as a pellet is retained, while the supernatant is discarded. The RBC pellet is washed and centrifuged thrice with phosphate-buffered saline (PBS, pH 7.4). After the final wash, the pellet was resuspended in PBS to prepare a 5% (*v*/*v*) RBC suspension. For the assay, 390 μL of the suspension is treated with 10 μL of the tested sample at varying concentrations and incubated for an hour without shaking. The final concentration of the tested compound in the final volume was 4 MIC, 2 MIC and 1 MIC. The drug was replaced with PBS, and 0.1% sodium dodecyl sulphate (SDS) served as the negative and positive control, respectively. After incubation, 100 μL of the test supernatant was carefully transferred to a 96-well microplate, and the absorbance was measured at 540 nm using the Victor3 microplate reader (Perkin Elmer, USA). All the experiments were performed in triplicate.

$$\%\;\mathrm{Hemolysis}=100\times \frac{\mathrm{ODsample}-\mathrm{ODpositive}\;\mathrm{control}}{\mathrm{ODpositive}\;\mathrm{control}-\mathrm{ODnegative}\;\mathrm{control}}$$

## 4. Conclusions

Chrysin and pinocembrin are key to the synergistic antifungal activity of propolis extract. This study attempted to improve the independent antimicrobial activity of chrysin and pinocembrin by synthesising their AA and TPP-based derivatives. AA conjugation improved the water solubility of the flavonoids and improved the antifungal activity of pinocembrin. The low activity of AA conjugates can be linked to their rapid hydrolysis in water. On the contrary, TPP-flavonoids with ether linkage showed promising antimicrobial activity with low hemolytic activity. The activity can be directly linked to the ability of the cationic TPP moiety to penetrate the cell, increasing the active delivery of the flavonoid into the cell. Further investigations established that the antifungal activity of the TPP-flavonoids is initiated by depolarisation of the mitochondrial membrane. Loss of membrane integrity and oxidative stress, which occur in later stages, also act as contributing factors to their antifungal mechanism. Hence, TPP derivatisation of flavonoids and other structurally similar molecules can be a potential method to improve their bioavailability and antimicrobial potency. The preliminary results obtained herein support further investigation of the synthesised TPP derivatives, particularly with regard to their antimicrobial potential and safety, including in vitro and in vivo cytotoxicity and toxicity studies.

## Data Availability

All the NMR spectra are attached in the Supplementary Materials.

## Supplementary Materials

The following supporting information can be downloaded at: https://www.mdpi.com/article/10.3390/antibiotics15101025/s1, Figure S1: The ^1^H NMR of compound **1**; Figure S2: The ^13^C NMR of compound **1**; Figure S3: The ^1^H NMR of compound **2**; Figure S4: The ^13^C NMR of compound **2**; Figure S5: The ^1^H NMR of compound **3**; Figure S6: The ^13^C NMR of compound **3**; Figure S7: The ^1^H NMR of compound **4**; Figure S8: The ^13^C NMR of compound **4**; Figure S9: The ^1^H NMR of compound **5**; Figure S10: The ^13^C NMR of compound **5**; Figure S11: The ^1^H NMR of compound **6**; Figure S12: The ^13^C NMR of compound **6**; Figure S13: The ^1^H NMR of compound **7**; Figure S14: The ^13^C NMR of compound **7**; Figure S15: The ^1^H NMR of compound **8**; Figure S16: The ^13^C NMR of compound **8**; Figure S17: The ^1^H NMR of compound **9**; Figure S18: The ^13^C NMR of compound **9**; Figure S19: The ^1^H NMR of compound **10**; Figure S20: The ^13^C NMR of compound **10**; Figure S21: The ^1^H NMR of compound **11**; Figure S22: The ^13^C NMR of compound **11**; Figure S23: The ^1^H NMR of compound **12**; Figure S24: The ^13^C NMR of compound **12**; Figure S25: Chromatograms of amino acid derivatives of chrysin indicative of hydrolysis to chrysin detectable with DAD at 267 nm: (a) Gly-Chrysin, (b) Leu-Chrysin, (c) Lys-Chrysin, (d) Pro-Chrysin and (e) Ser-Chrysin. The retention time and peak area are labelled with *; Figure S26: Chromatograms of amino acid derivatives of pinocembrin indicative of hydrolysis to pinocembrin detectable with DAD at 267 nm: (a) Gly- Pinocembrin, (b) Leu- Pinocembrin, (c) Lys- Pinocembrin, (d) Pro- Pinocembrin and (e) Ser- Pinocembrin. The retention time and peak area are labelled with *; Figure S27: Optical density of AA conjugates in fungal medium (RPMI) and bacterial medium (MHB), measured at equimolar concentrations corresponding to 1024 µg/mL of their respective parent molecules. Compounds **1**–**5** are chrysin-based conjugates, while compounds **6**–**10** are pinocembrin-based conjugates.

## Abbreviations

The following abbreviations are used in this manuscript:

ACN	acetonitrile
AcOEt	ethyl acetate
Boc	*tert*-butoxycarbonyl group
CHR	chrysin
DCHA	dicyclohexylamine
DMAP	4-(dimethylamino)pyridine
DMSO	dimethyl sulfoxide
EDCI	1-ethyl-3-(3-dimethylaminopropyl)carbodiimide hydrochloride
Et_2_O	diethyl ether
H_2_-DCFDA	2′,7′-dichlorodihydrofluorescein diacetate
iPrOH	isopropanol
MeOH	methanol
MIC	minimum inhibitory concentration
PI	propidium iodide
PIN	pinocembrin
Rh123	rhodamine 123
ROS	reactive oxygen species
TFA	trifluoroacetic acid
TPP	triphenylphosphonium cation
