# Supplementary material for: Investigation of Antimicrobial Activity of Amino Acid- and Triphenylphosphonium-Based Derivatives of Chrysin and Pinocembrin
Source: Antibiotics (Basel). 2026 Oct 15;15(10):1025. doi: 10.3390/antibiotics15101025 (PMC13700714; doi:10.3390/antibiotics15101025)
Supplement: Supplementary file 1 [file antibiotics-15-01025-s001.zip › antibiotics-4589047-supplementary.pdf]

# Investigation of Antimicrobial Activity of Amino Acid- and Triphenylphosphonium-Based Derivatives of Chrysin and Pinocembrin

Jeslin Cheruathoor Jenny,[a] Andrzej S. Skwarecki,[a]* Michał K. Pierański,[a] Katarzyna Kozłowska-Tylingo,[a] Piotr Szweda[a*]

[a]Department of Pharmaceutical Technology and Biochemistry, Gdańsk University of Technology, 11/12 Gabriela Narutowicza Street, 80-233 Gdańsk, Poland

*Correspondence: Piotr Szweda: piotr.szweda@pg.edu.pl
Andrzej S. Skwarecki: andrzej.skwarecki1@pg.edu.pl

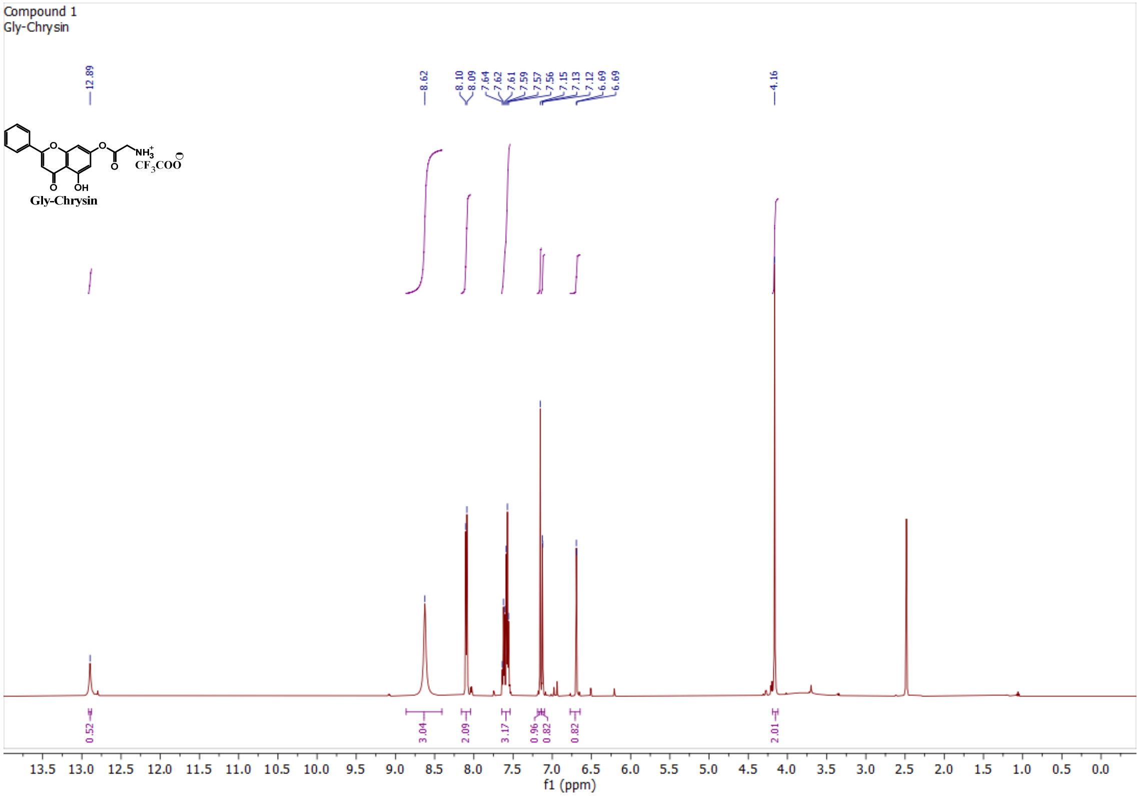


Figure S1. The $^1$H NMR of compound 1.

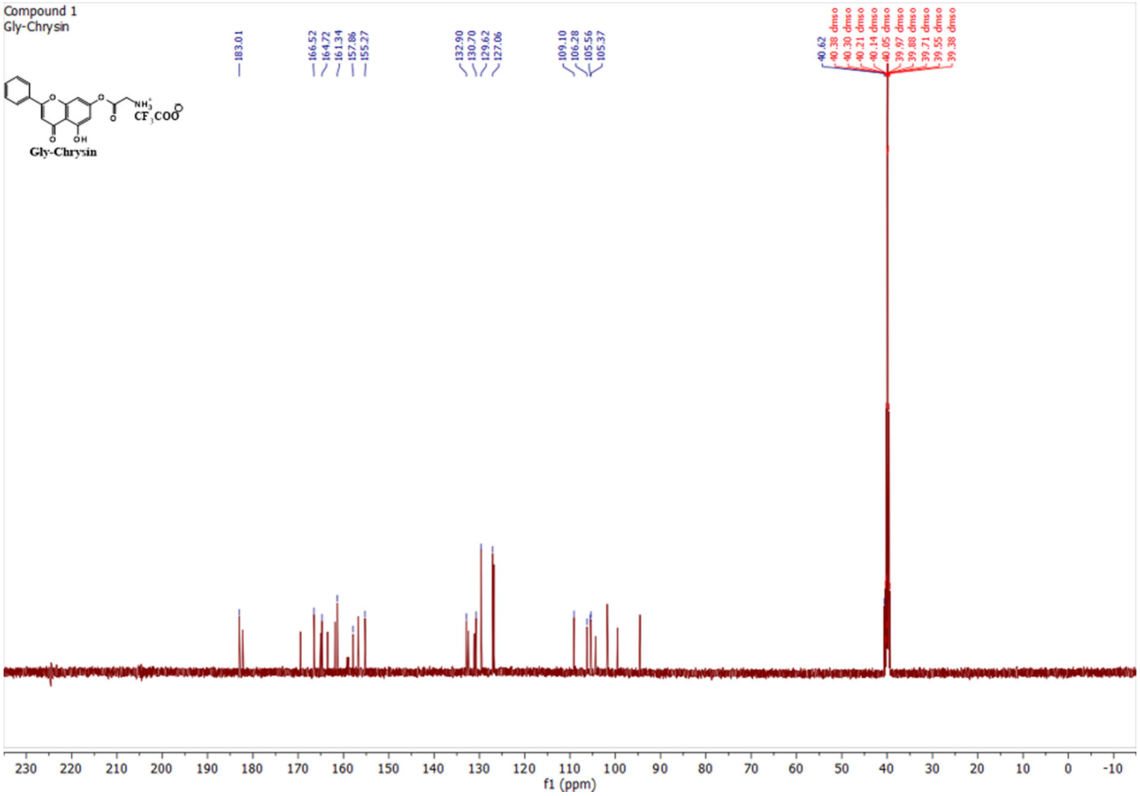


Figure S2. The $^{13}C$ NMR of compound 1.

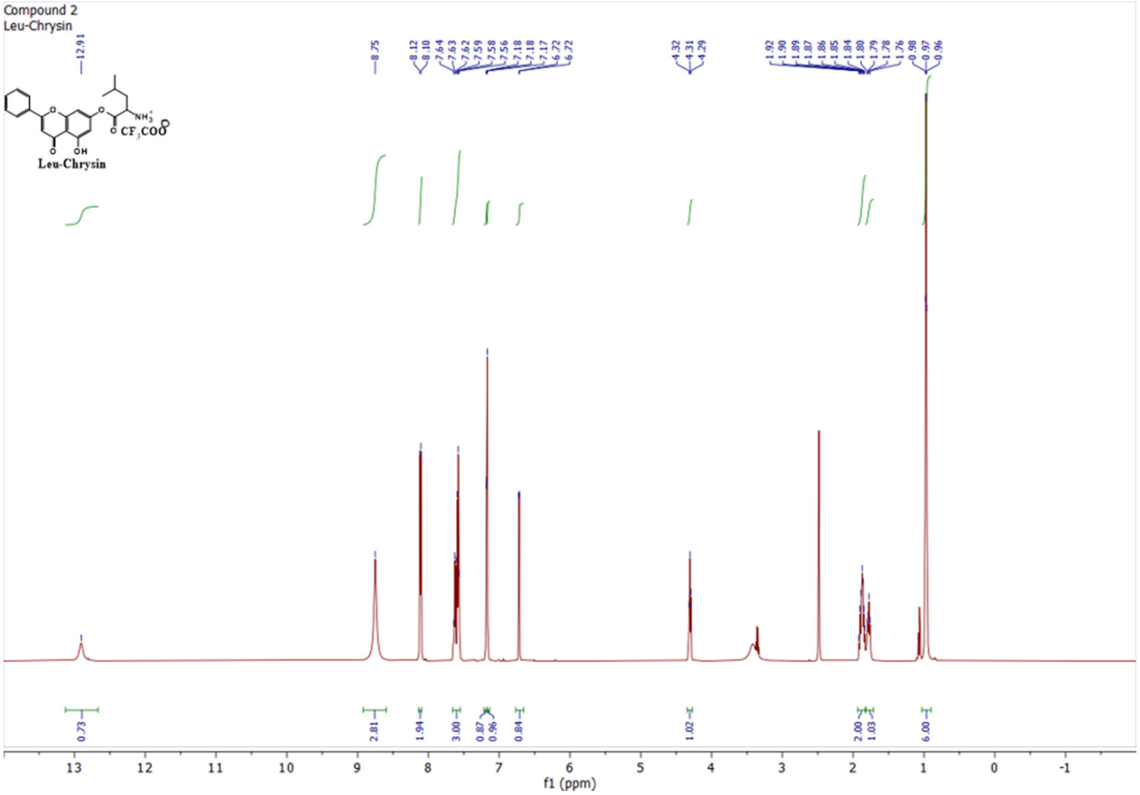


Figure S3. The $^{1}H$ NMR of compound 2.

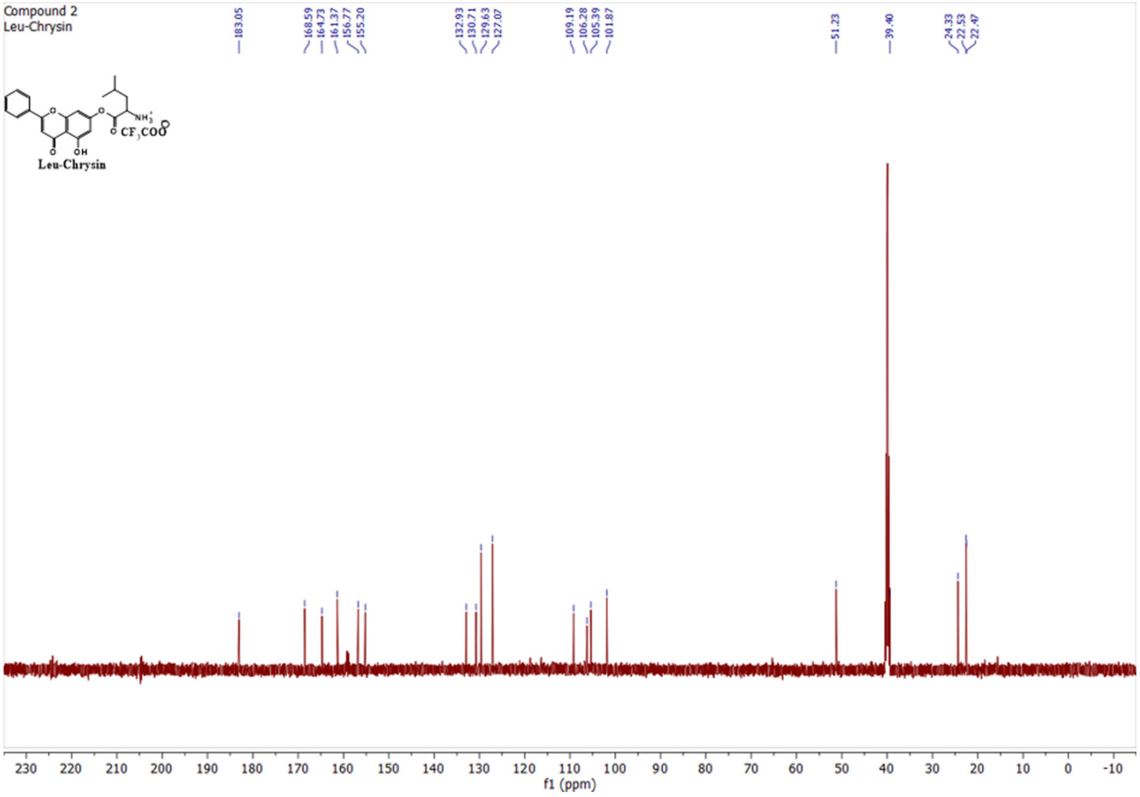


Figure S4. The $^{13}$C NMR of compound 2.

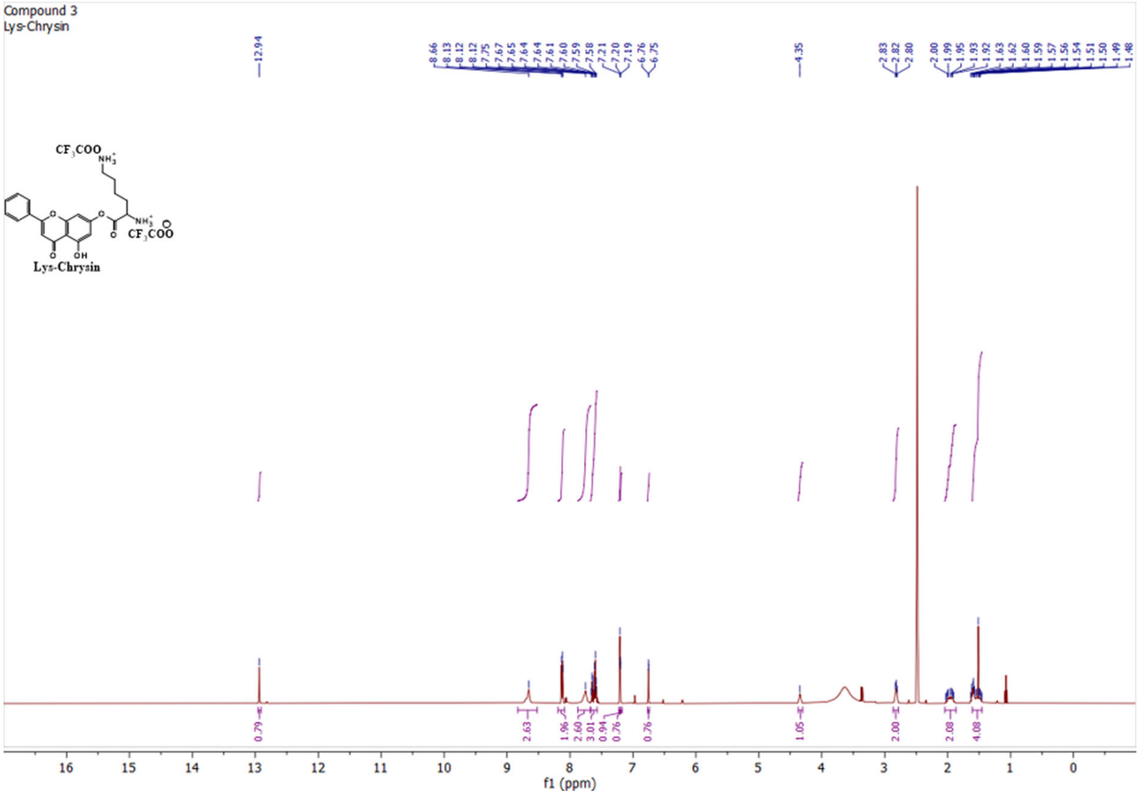


Figure S5. The $^{1}$H NMR of compound 3.

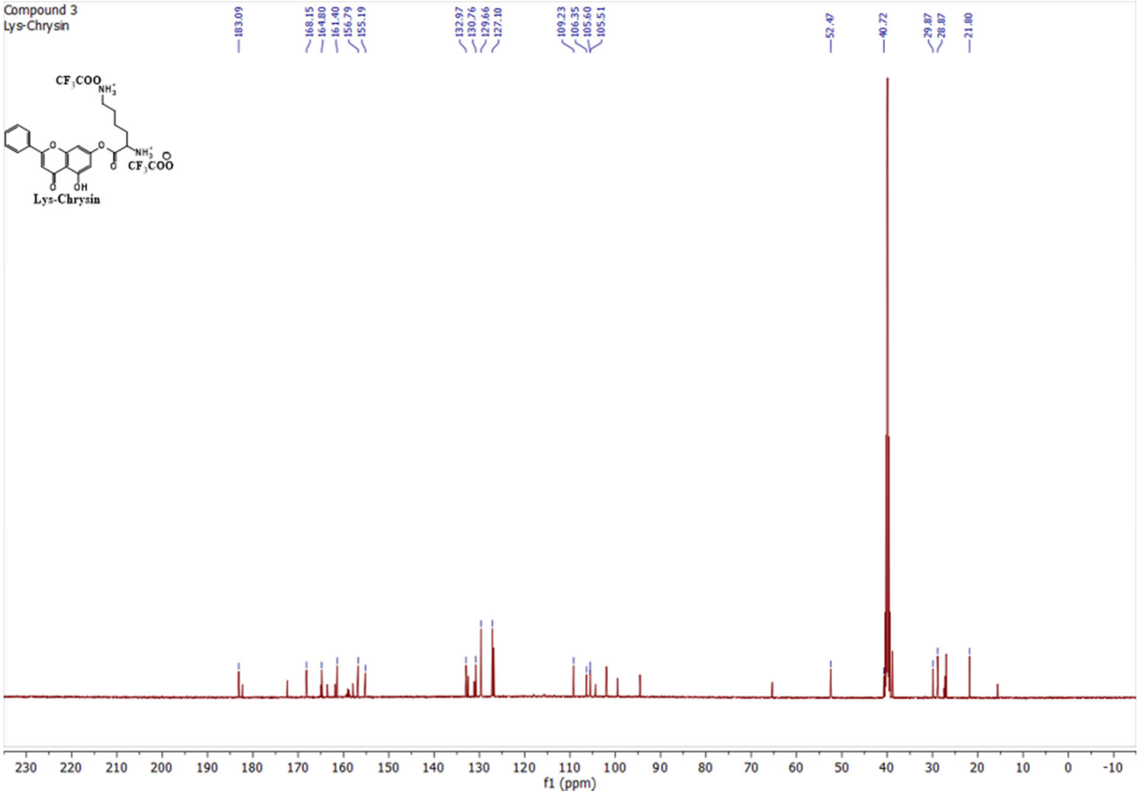


Figure S6. The $^{13}$C NMR of compound 3.

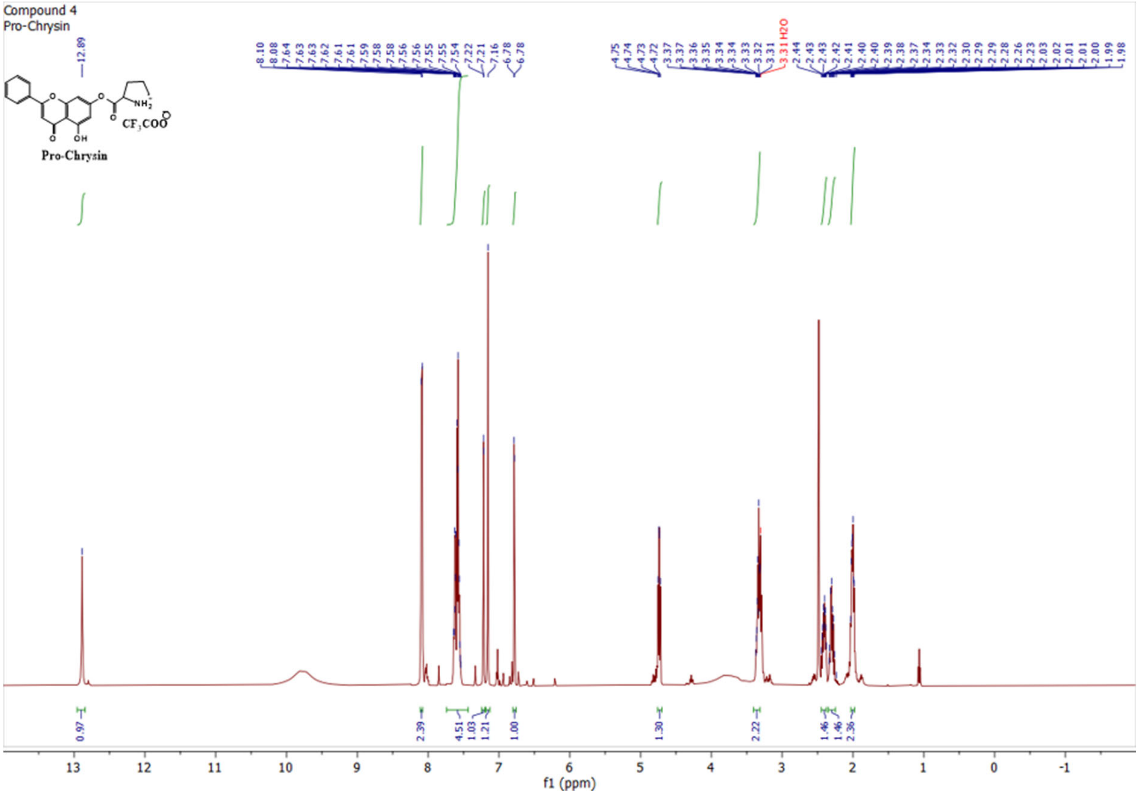


Figure S7. The $^{1}$H NMR of compound 4.

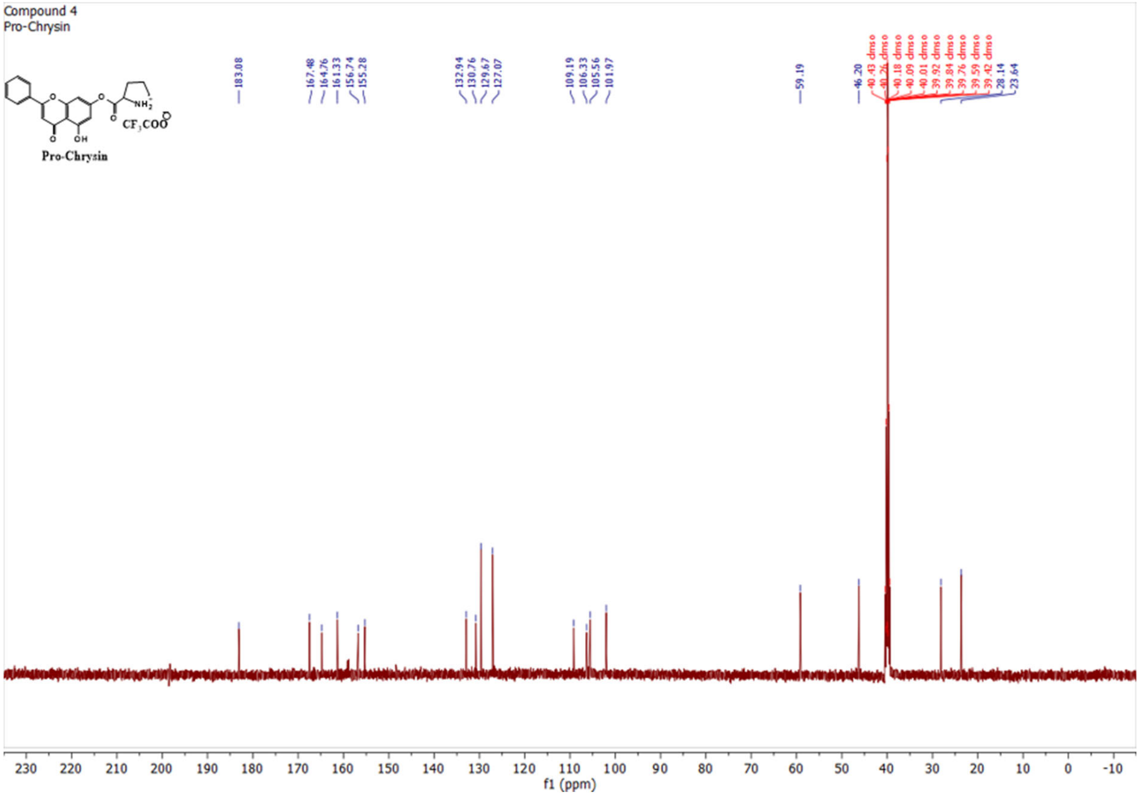


Figure S8. The $^{13}$C NMR of compound 4.

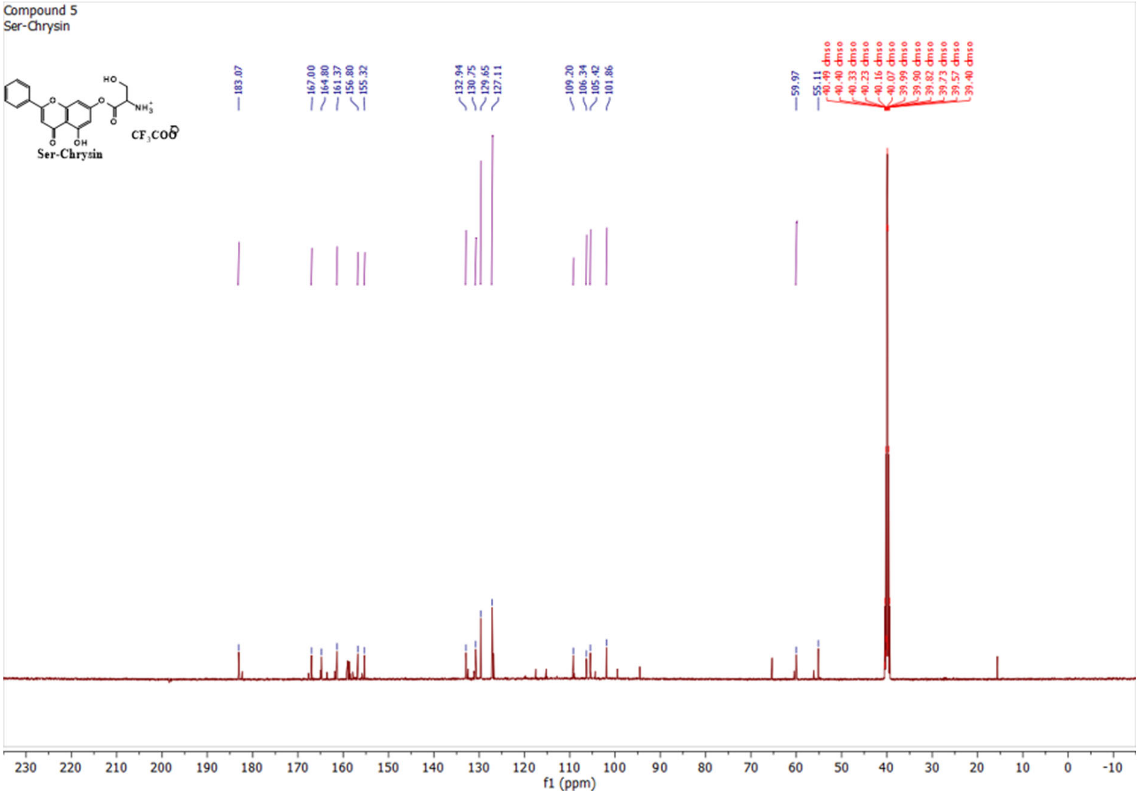


Figure S9. The $^{1}$H NMR of compound 5.

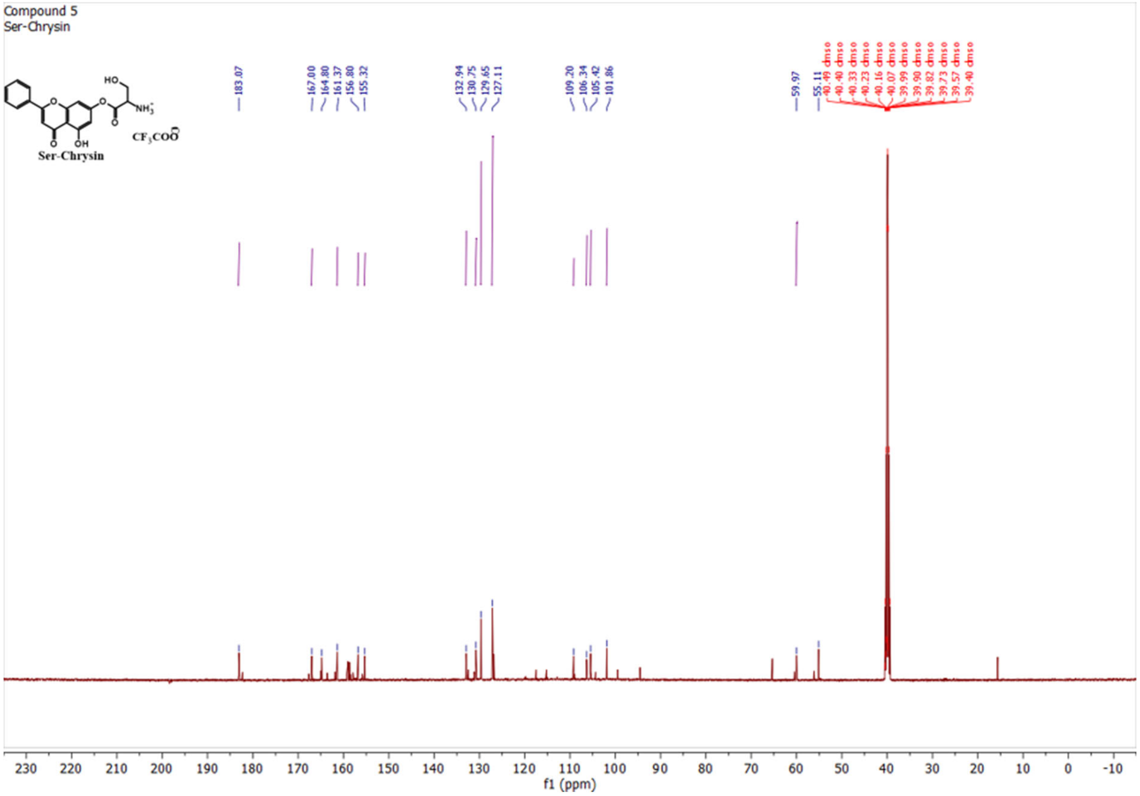


Figure S10. The $^{13}C$ NMR of compound 5.

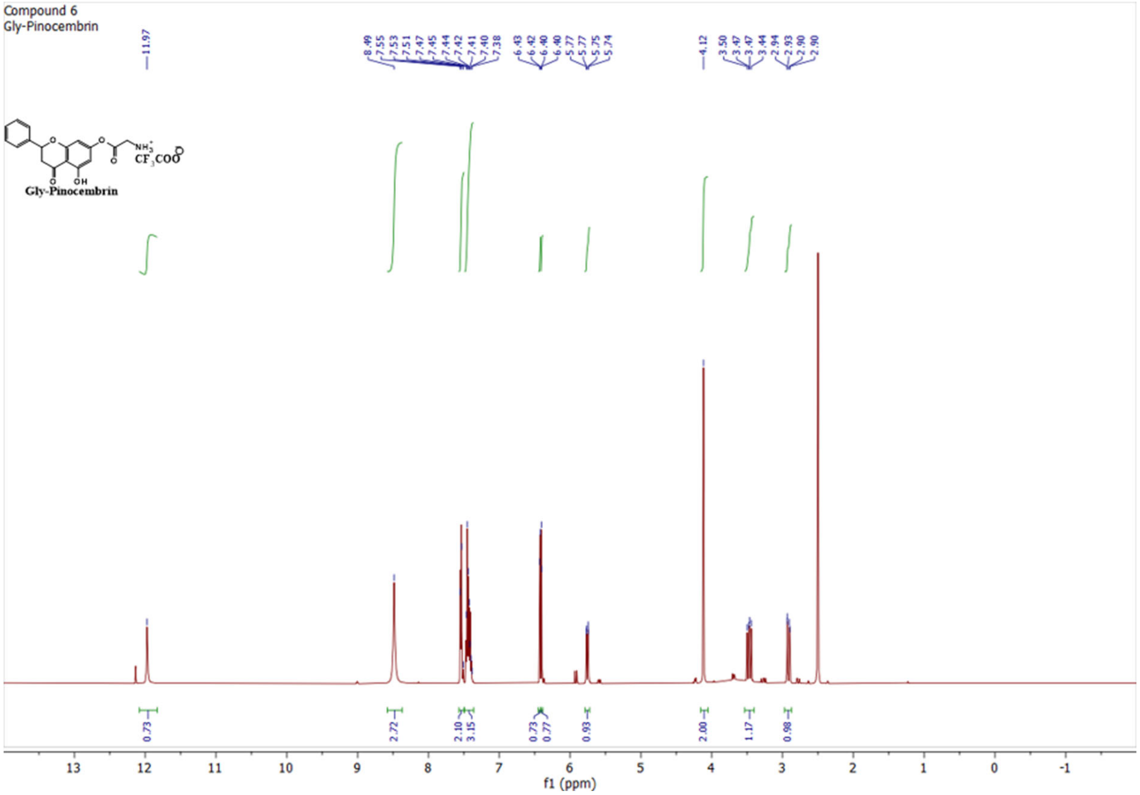


Figure S11. The $^{1}H$ NMR of compound 6.

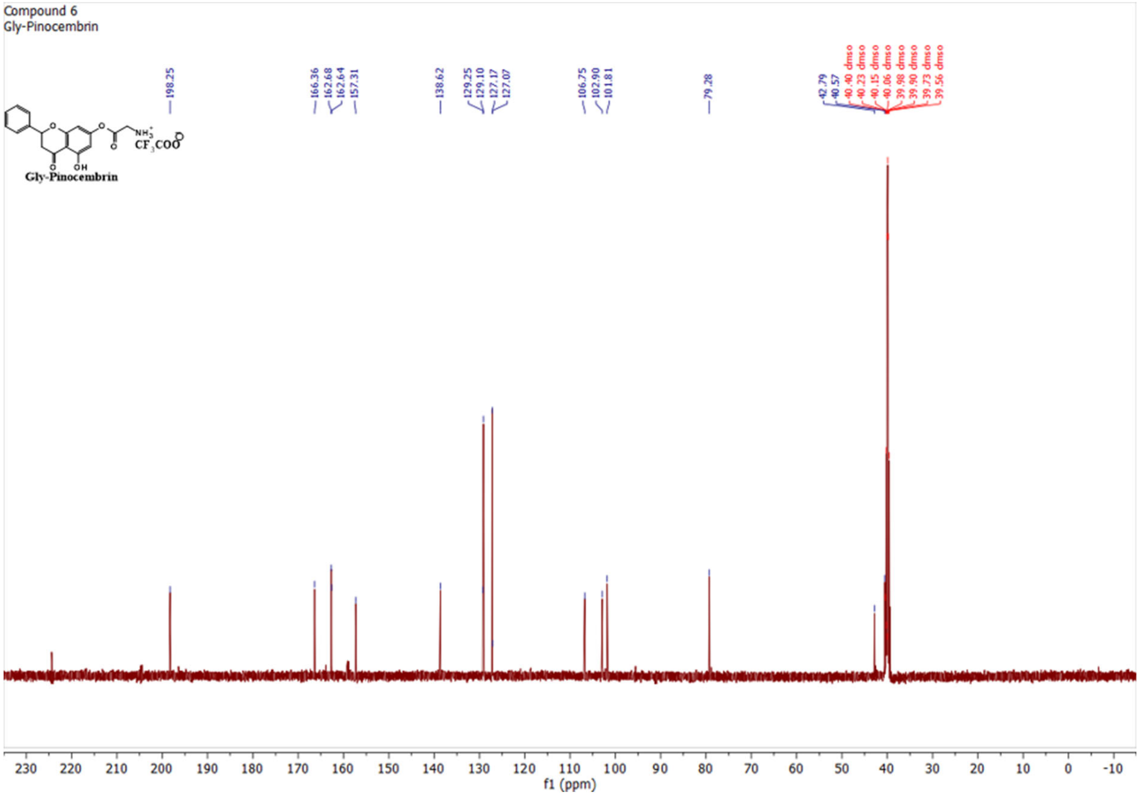


Figure S12. The $^{13}$C NMR of compound 6.

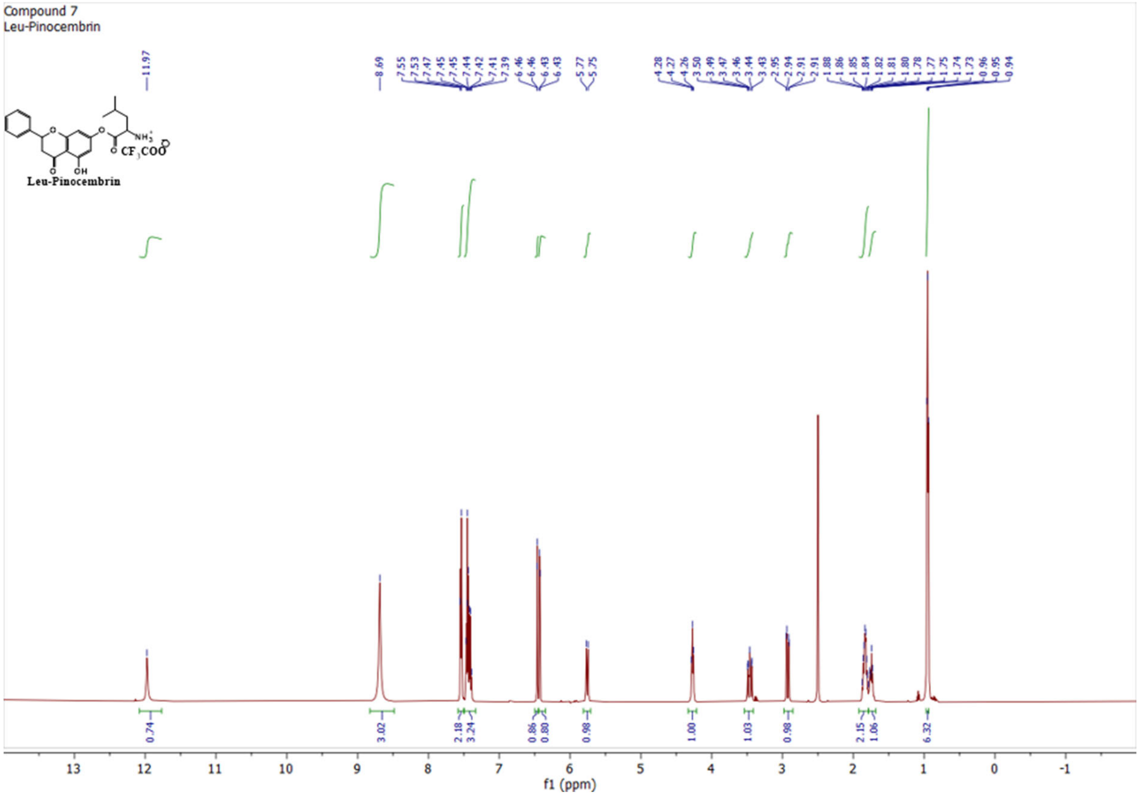


Figure S13. The $^{1}$H NMR of compound 7.

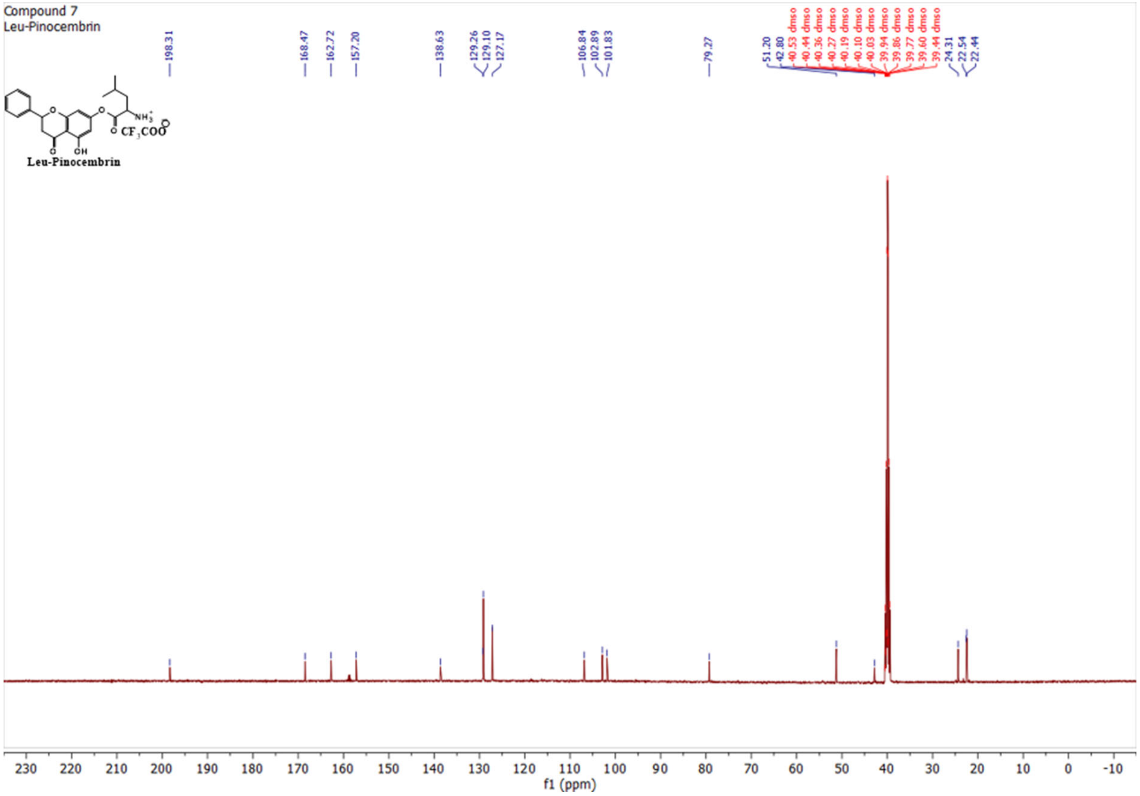


Figure S14. The $^{13}$C NMR of compound 7.

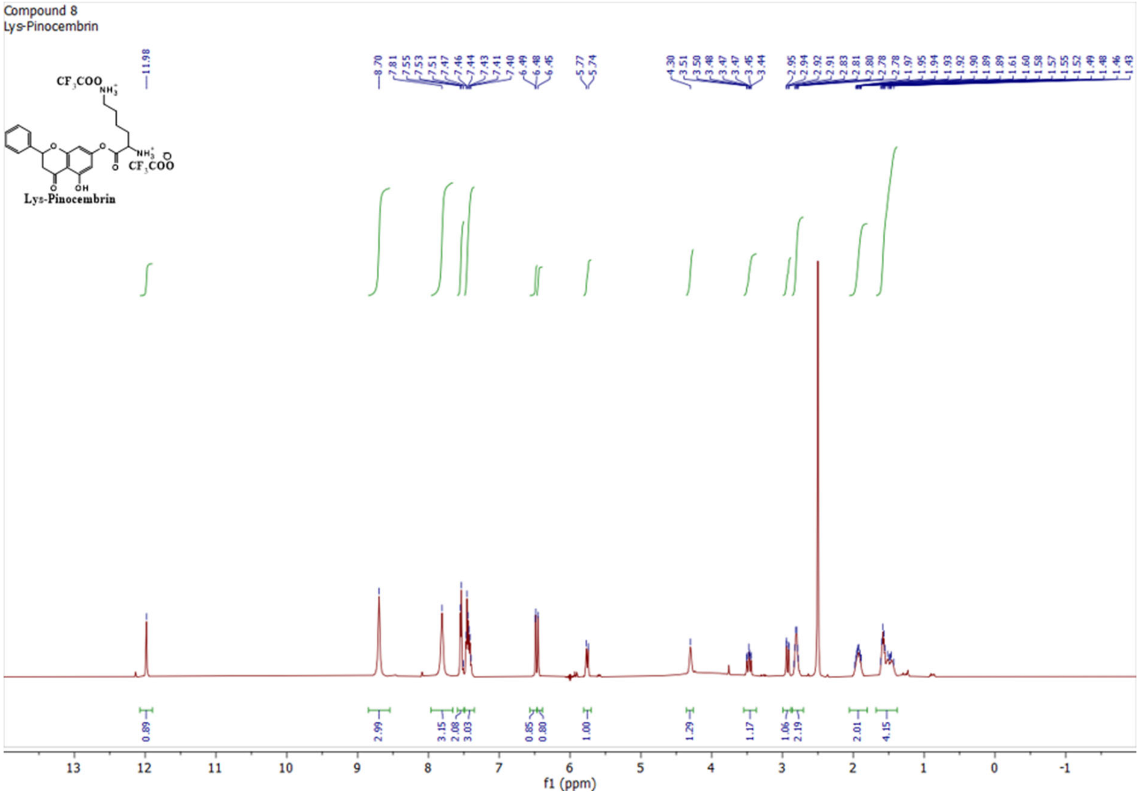

Figure S15. The $^{1}$H NMR of compound 8

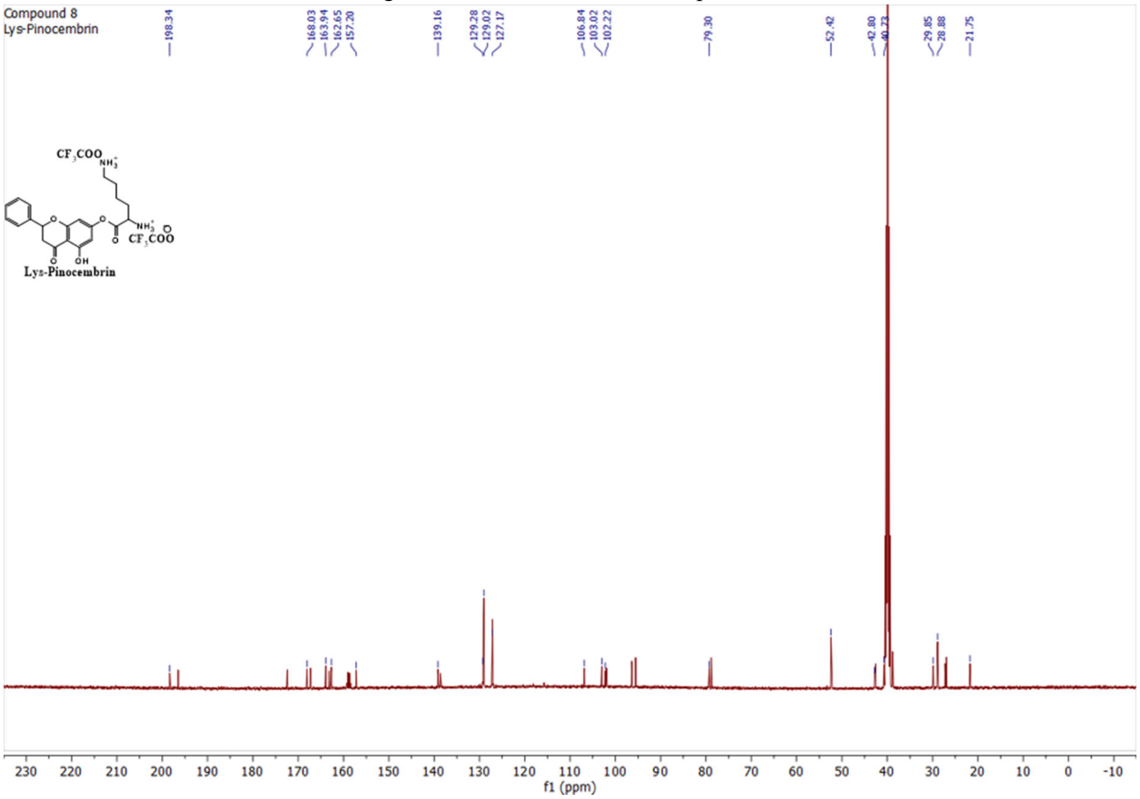


Figure S16. The $^{13}$C NMR of compound 8.

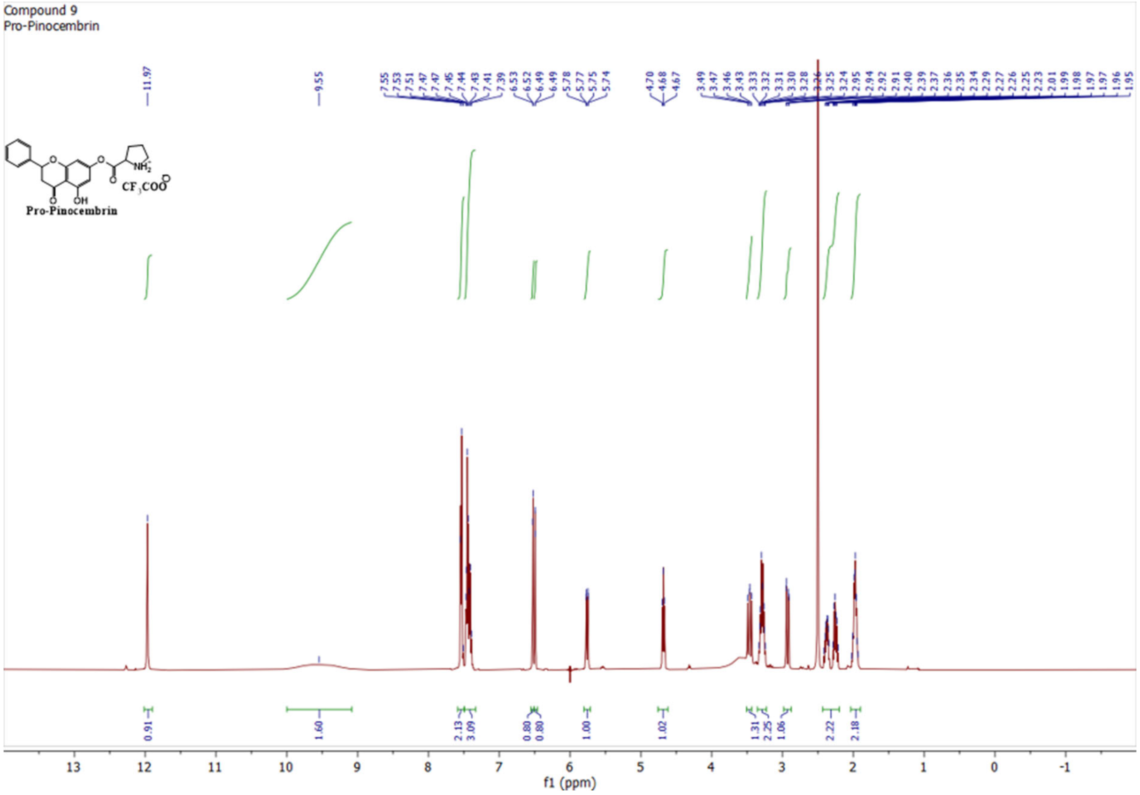


Figure S17. The $^{1}$H NMR of compound 9.

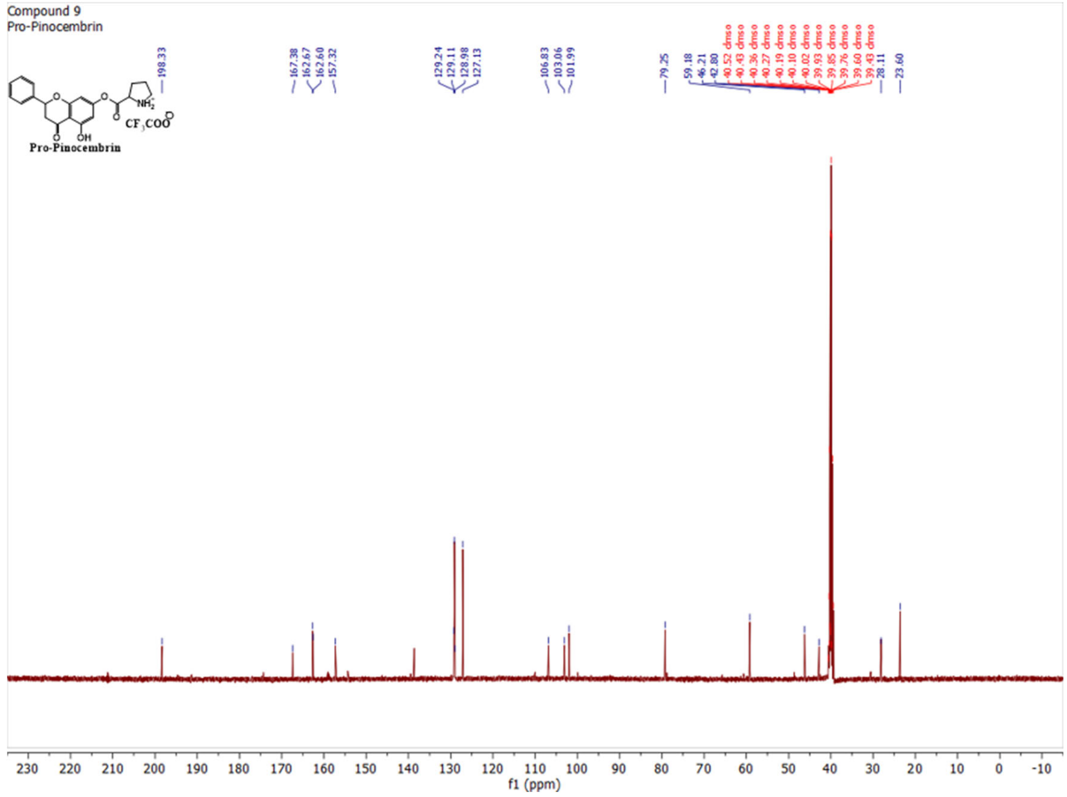


Figure S18. The $^{13}C$ NMR of compound 9.

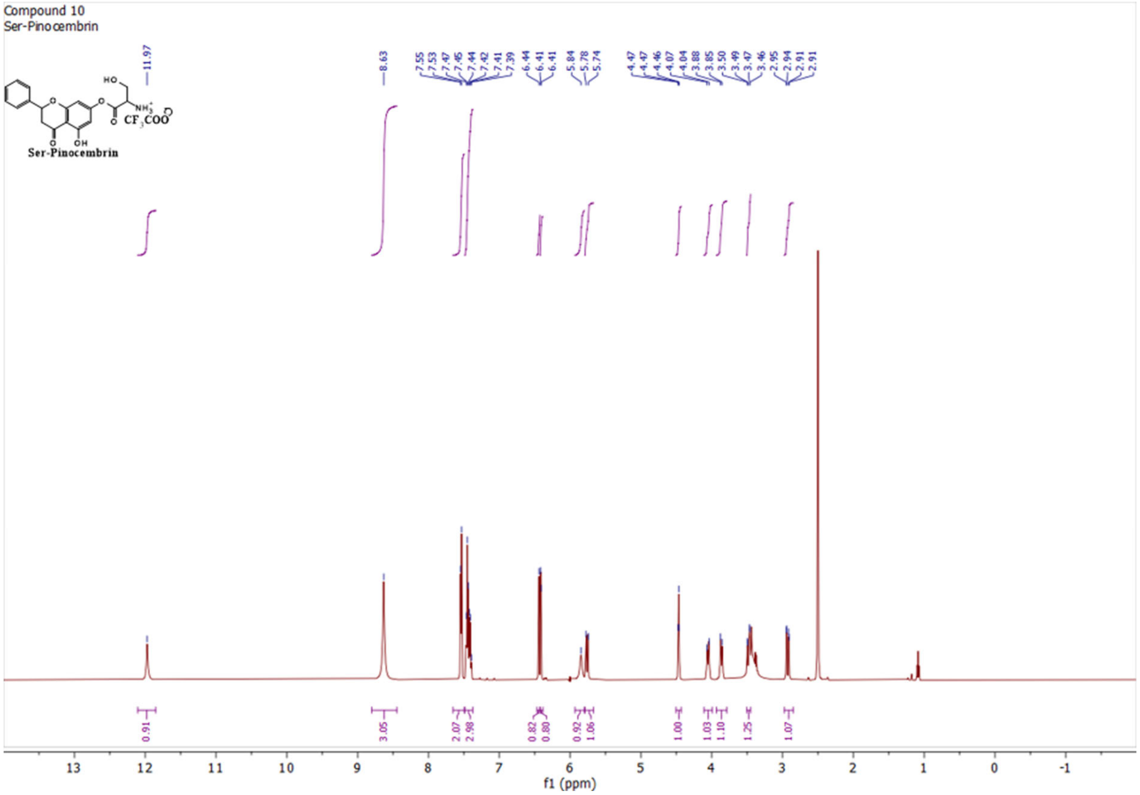


Figure S19. The $^{1}H$ NMR of compound 10.

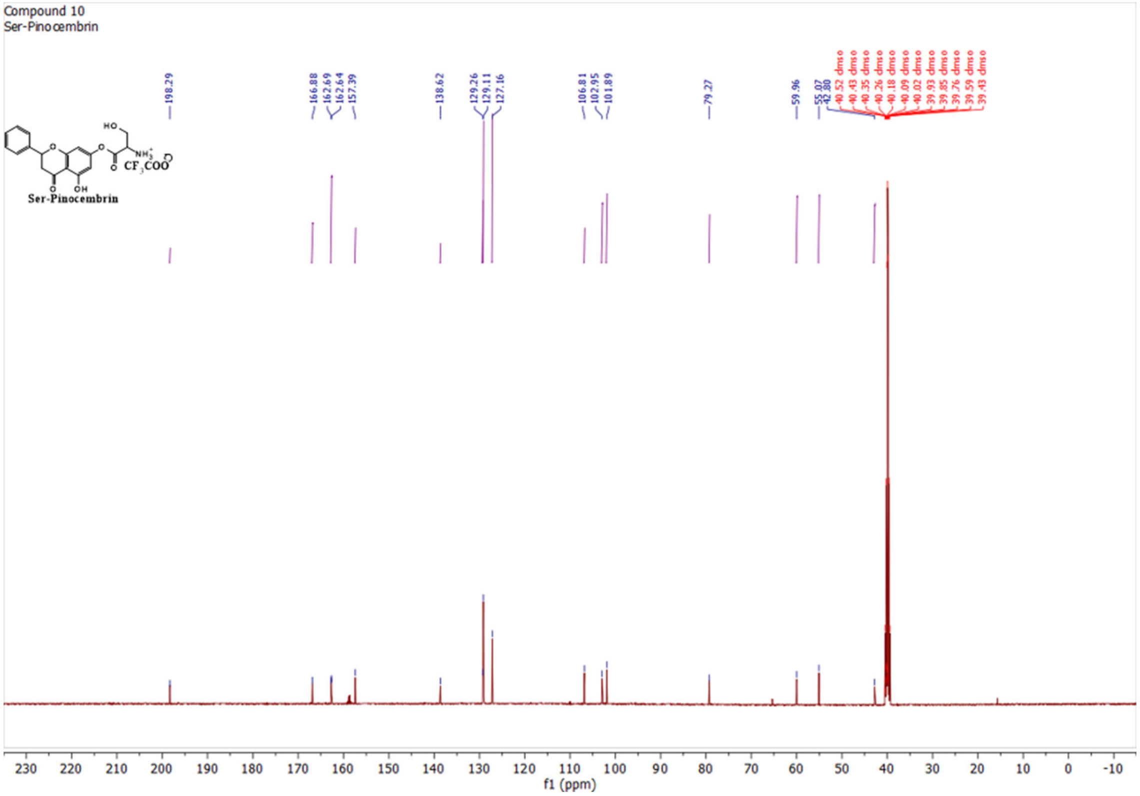

Figure S20. The $^{13}C$ NMR of compound 10.

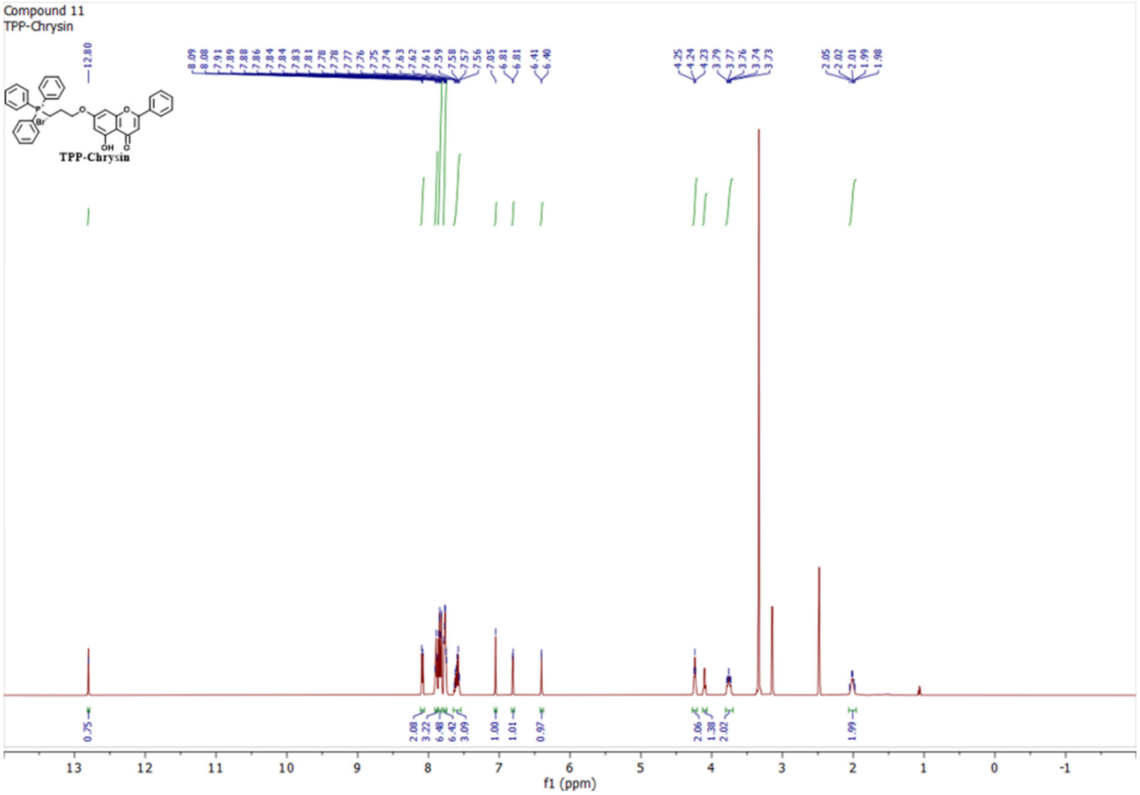

Figure S21. The $^{1}H$ NMR of compound 11.

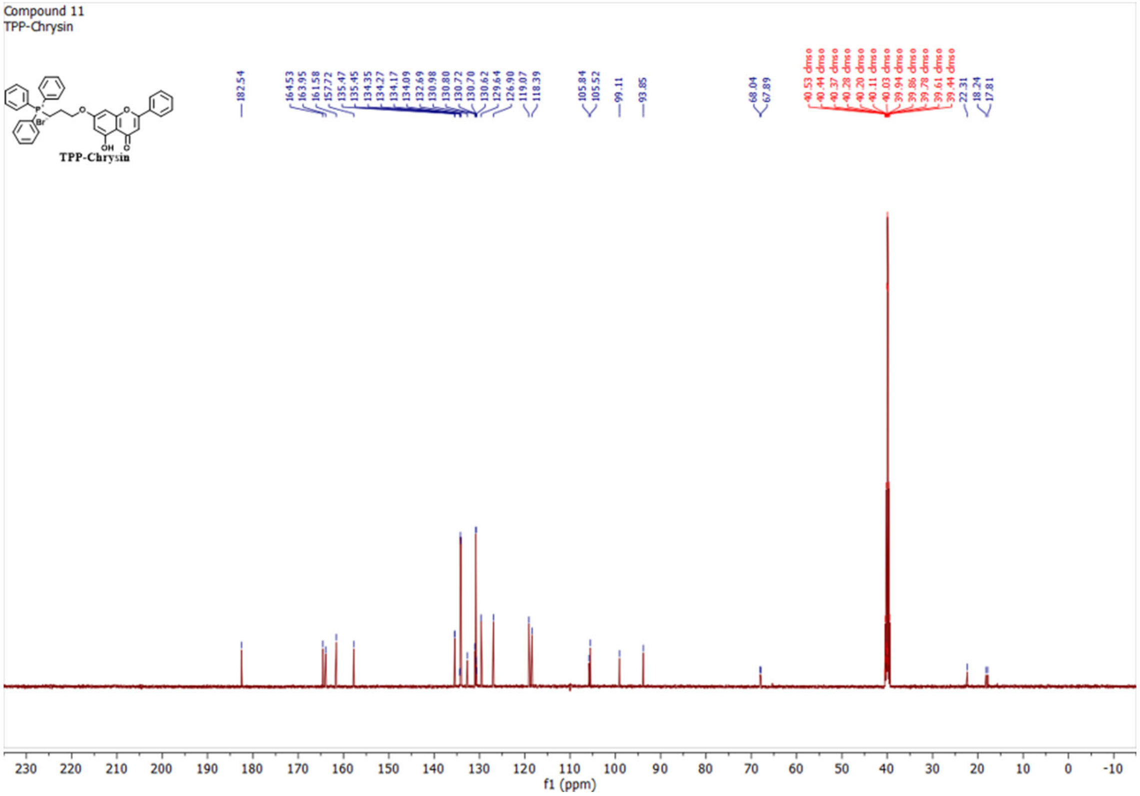


Figure S22. The $^{13}$C NMR of compound 11.

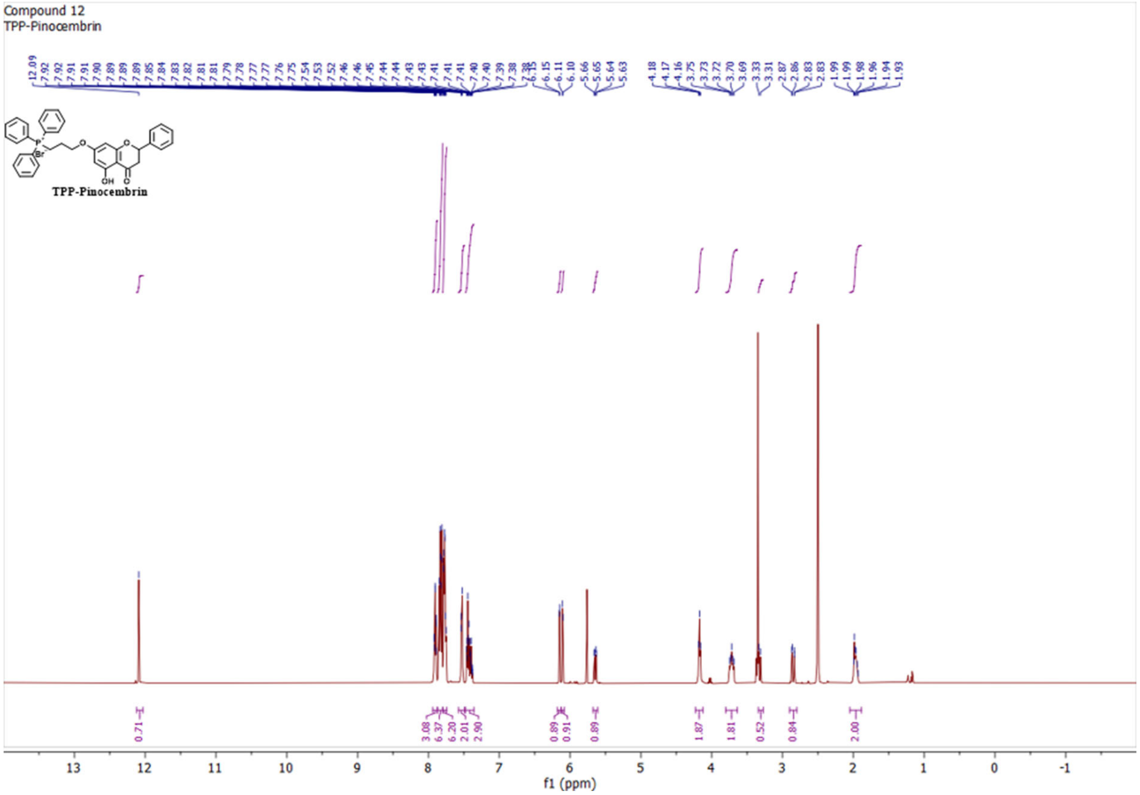


Figure S23. The $^{1}$H NMR of compound 12.

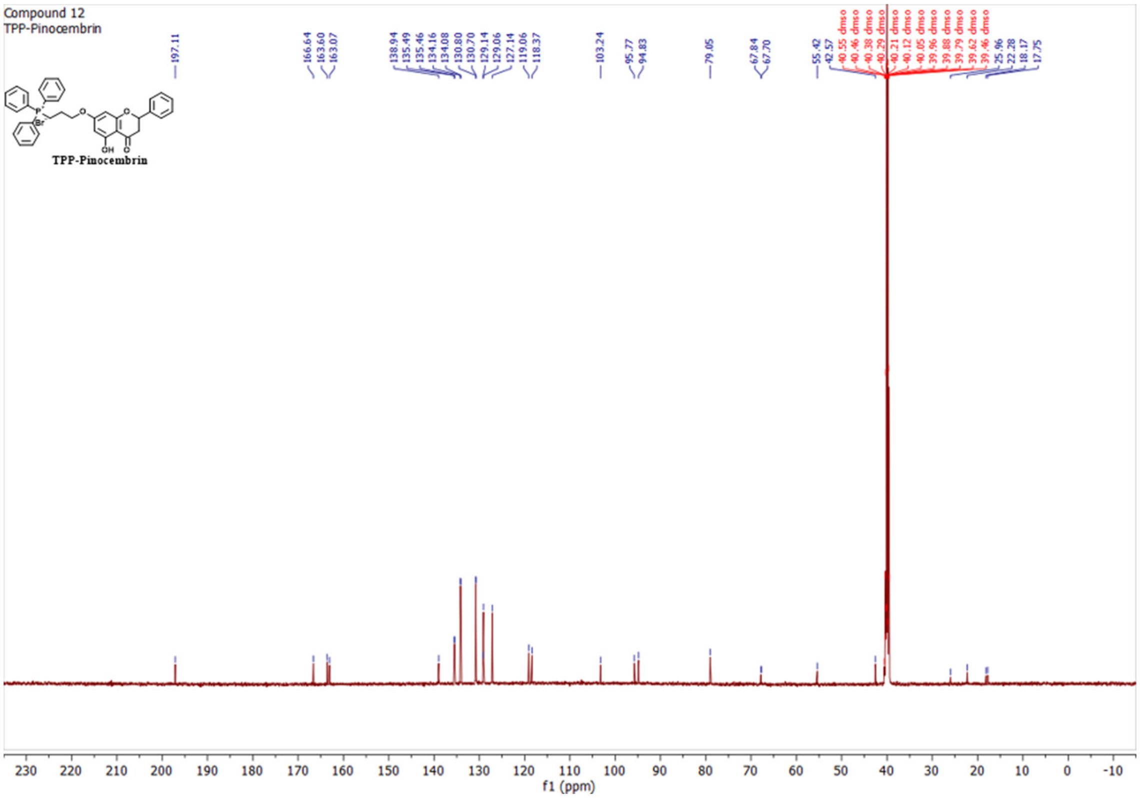


Figure S24. The $^{13}C$ NMR of compound 12.

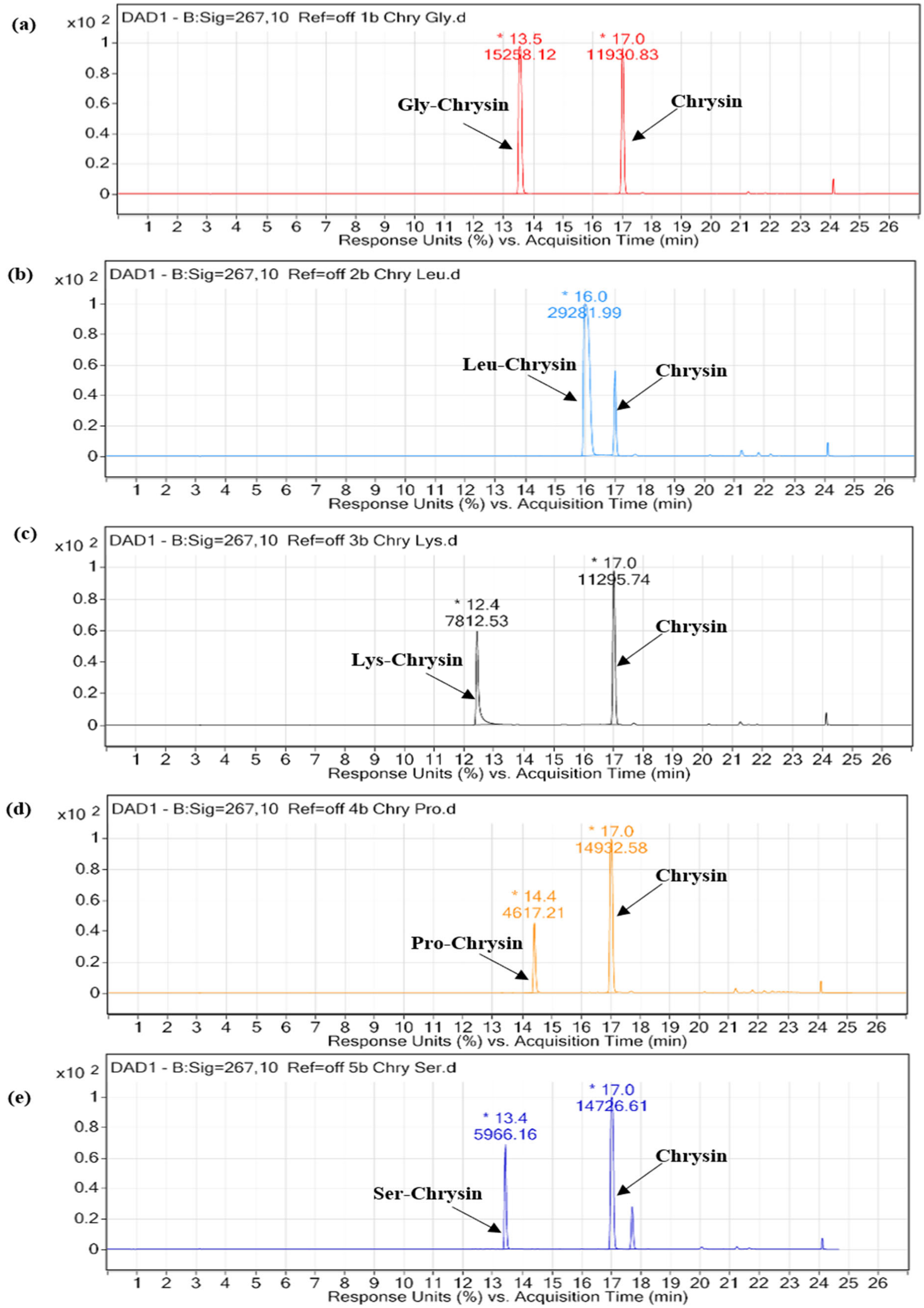


Figure S25: Chromatograms of amino acid derivatives of chrysin indicative of hydrolysis to chrysin detectable with DAD at 267 nm: (a) Gly-Chrysin, (b) Leu-Chrysin, (c) Lys-Chrysin, (d) Pro-Chrysin and (e) Ser-Chrysin. The retention time and peak area are labelled with *.

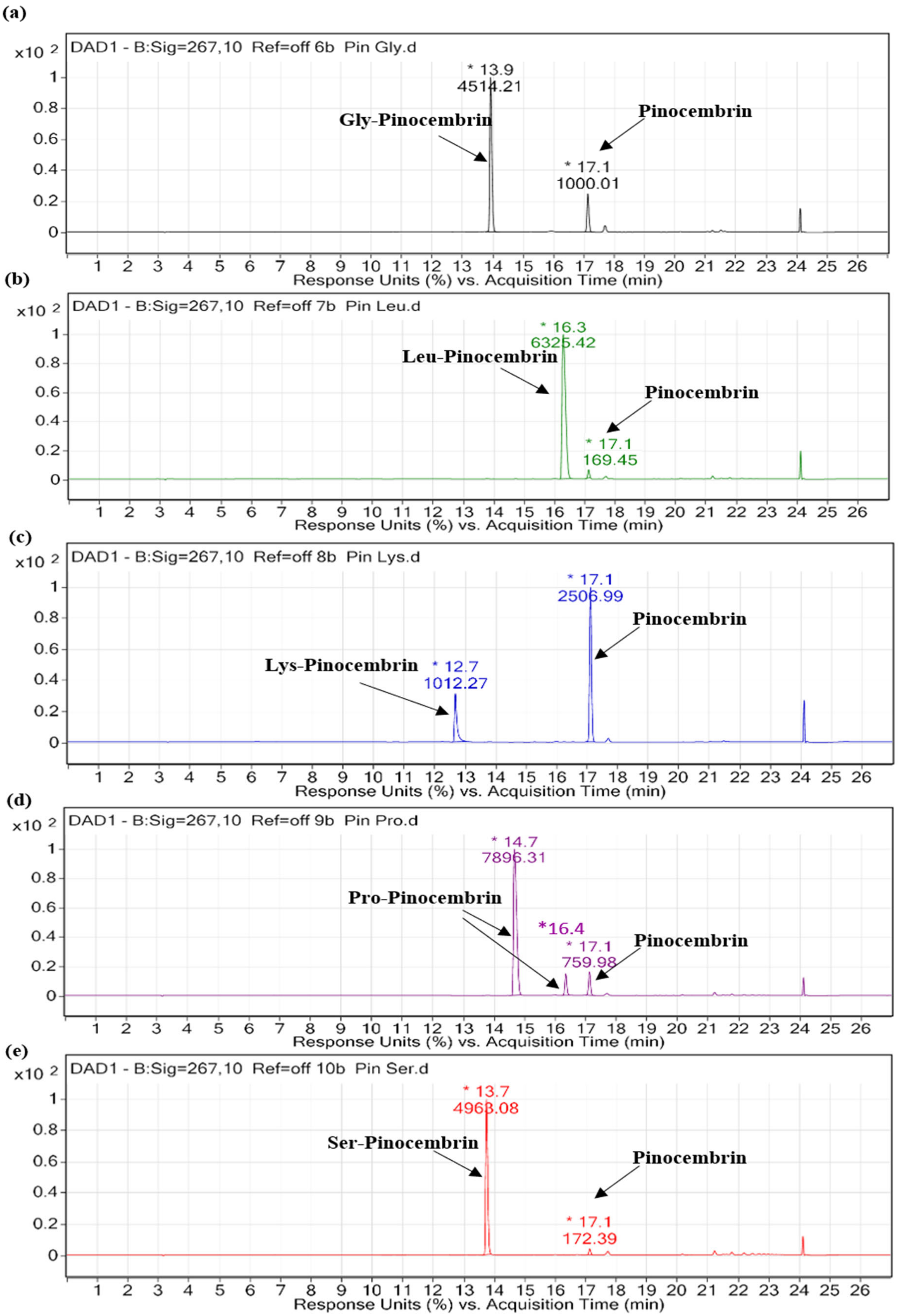


Figure S26: Chromatograms of amino acid derivatives of pinocembrin indicative of hydrolysis to pinocembrin detectable with DAD at 267 nm: (a) Gly- Pinocembrin, (b) Leu- Pinocembrin, (c) Lys- Pinocembrin, (d) Pro-Pinocembrin and (e) Ser- Pinocembrin. The retention time and peak area are labelled with *.

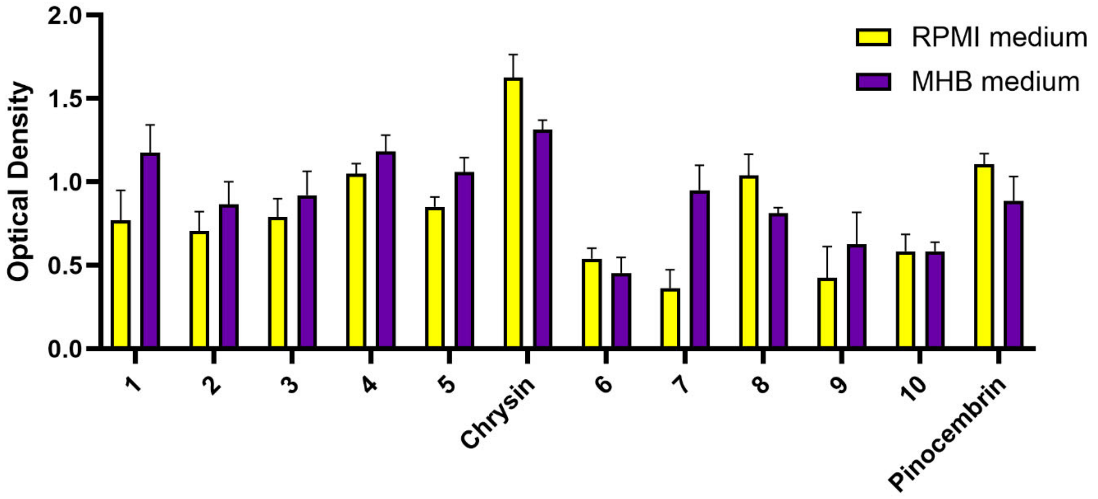

Figure S27: Optical density of AA conjugates in fungal medium (RPMI) and bacterial medium (MHB), measured at equimolar concentrations corresponding to 1024 µg/mL of their respective parent molecules. Compounds 1–5 are chrysin-based conjugates, while compounds 6–10 are pinocembrin-based conjugates.
